# Accumulation of blood-circulating PD-L1-expressing M-MDSCs and monocytes/macrophages in pretreatment ovarian cancer patients is associated with soluble PD-L1

**DOI:** 10.1186/s12967-020-02389-7

**Published:** 2020-06-01

**Authors:** Karolina Okła, Alicja Rajtak, Arkadiusz Czerwonka, Marcin Bobiński, Anna Wawruszak, Rafał Tarkowski, Wiesława Bednarek, Justyna Szumiło, Jan Kotarski

**Affiliations:** 1 The First Department of Oncologic Gynecology and Gynecology, Medical University of Lublin, 20-081 Lublin, Poland; 2 Department of Virology and Immunology, Maria Curie-Sklodowska University, 20-031, Lublin, Poland; 3 Department of Biochemistry and Molecular Biology, Medical University of Lublin, 20-081 Lublin, Poland; 4 Department of Clinical Pathomorphology, Medical University of Lublin, 20-090 Lublin, Poland

**Keywords:** Ovarian cancer, M-MDSC, Monocytes/macrophages, PD-L1, sPD-L1, TMEs, Immunosuppression, Liquid biopsy

## Abstract

**Background:**

Previous studies have shown clinical relevance of programmed death-ligand 1 (PD-L1) and soluble PD-L1 (sPD-L1) in human cancers. However, still contradictory results exist. Our aim was evaluation of PD-L1-expressing monocytic myeloid-derived suppressor cells (M-MDSCs), monocytes/macrophages (MO/MA), tumour cells (TC) and immune/inflammatory cells (IC) as well as investigation of the sPD-L1 in ovarian cancer (OC) patients.

**Methods:**

The group of 74 pretreatment women were enrollment to the study. The expression of PD-L1 on M-MDSCS and MO/MA was assessed by flow cytometry. The profile of sPD-L1 was examined with ELISA. The expression of PD-L1 in mononuclear cells (MCs) was analyzed using real time PCR. PD-L1 immunohistochemical analysis was prepared on TC and IC. An in silico validation of prognostic significance of PD-L1 mRNA expression was performed based microarray datasets.

**Results:**

OC patients had significantly higher frequency of MO/MA versus M-MDSC in the blood, ascites and tumour (each p < 0.0001). In contrast, PD-L1 expression was higher on M-MDSCs versus MO/MA in the blood and ascites (each p < 0.0001), but not in the tumour (p > 0.05). Significantly higher accumulation of blood-circulating M-MDSC, MO/MA, PD-L1^+^M-MDSC, PD-L1^+^MO/MA and sPD-L1 was observed in patients versus control (p < 0.001, p < 0.05, p < 0.001, p < 0.001 and p < 0.0001, respectively). Accumulation of these factors was clinicopathologic-independent (p > 0.05). The expression of PD-L1 was significantly higher on IC versus TC (p < 0.0001) and was clinicopathologic-independent (p > 0.05) except higher level of PD-L1^+^TC in the endometrioid versus mucinous tumours. Interestingly, blood-circulating sPD-L1 positively correlated with PD-L1^+^M-MDSCs (p = 0.03) and PD-L1^+^MO/MA (p = 0.02) in the blood but not with these cells in the ascites and tumours nor with PD-L1^+^TC/IC (each p > 0.05). PD-L1 and sPD-L1 were not predictors of overall survival (OS; each p > 0.05). Further validation revealed no association between PD-L1 mRNA expression and OS in large independent OC patient cohort (n = 655, p > 0.05).

**Conclusions:**

Although PD-L1 may not be a prognostic factor for OC, our study demonstrated impaired immunity manifested by up-regulation of PD-L1/sPD-L1. Furthermore, there was a positive association between PD-L1^+^ myeloid cells and sPD-L1 in the blood, suggesting that sPD-L1 may be a noninvasive surrogate marker for PD-L1^+^myeloid cells immunomonitoring in OC. Overall, these data should be under consideration during future clinical studies/trials.

## Background

Among gynecological malignancies, ovarian cancer (OC) is the prominent cause of death due to its predominantly advanced stage at diagnosis. It has been reported that over the past 30 years, all cancers showed 20% increase in the 5-year survival. Despite this, survival rate for OC has been almost the same for decades, and remains at only 47% in 5 years after diagnosis; by comparison, in breast cancer 5-year survival rate is 85% [1]. Although standard care for patients including surgical cytoreduction followed by chemotherapy is often satisfactory, recurrence frequently occurs, and relapse tumours respond poorly to currently available treatment. Ovarian cancers are immunogenic tumours which can induce antitumour immune response found in different tumour microenvironments (TMEs) i.e. blood, ascites and tumour tissue. Notwithstanding, there is growing body of evidence presented by our and other research groups about the dynamic and complex immunosuppressive network in the TMEs of human ovarian cancer [2–5]. The barrier presented by immunosuppression in the ovarian TMEs lead to disappointing results of immunotherapy and is one of the biggest challenges for successful immunotherapy to prevent recurrence of disease and progression after debulking surgery and chemotherapy.

In ovarian cancer, inflammatory myeloid cells i.e. monocytes/macrophages (MO/MA) are mainly immunosuppressive, and promote invasion, angiogenesis, metastasis and recurrence of tumour. Furthermore, immature myeloid cells e.g. monocytic-myeloid-derived suppressor cells (M-MDSCs) play an important role in strengthening the disease, increasing tumour burden, and resisting to (immuno)therapy in ovarian cancer [5]. MDSCs can inhibit the antitumour reactivity using the broad spectrum of immunosuppressive factors including mainly the production of arginase 1 (ARG1), indoleamine 2,3-dioxygenase (IDO), interleukin 10 (IL-10), transforming growth factor β (TGF-β) as well as increased expression of programmed death-ligand 1 (PD-L1). This immunosuppressive activity of MDSCs can significantly promote both tumour growth and metastasis and finally influence on clinical manifestation of the disease [6]. M-MDSCs and monocytes/macrophages can be separated based on expression of major histocompatibility complex (MHC) class II molecules. M-MDSC has a phenotype HLA-DR^−/low^CD14^+^, whereas MO/MA are HLA-DR^+^CD14^+^ cells [7].

Although cancer cells use myriad arsenal of immunosuppressive mechanisms to escape from the host immune system, PD-L1 (also known as CD274 or B7-H1), seems to be one of the hallmarks of a progressive tumour which can hamper (immuno)therapy efficacy [8]. PD-L1 can be expressed in both tumour cells and immune/inflammatory cells and its expression can be associated with poor prognosis in many human malignancies [9]. Although immune checkpoint inhibitors of PD-L1 has shown improving survival rates in some patients with malignancies effectiveness of PD-L1 blockade is unsatisfactory (response rate is only ~ 20–30%) [10] and still many factors affecting this effectiveness are unknown. The reversing of immunosuppressive TMEs of ovarian cancer and sensitize these tumours to immunotherapeutics can be a “Holy Grail” in the cancer treatment.

Growing body of evidence support clinical utility of immunological markers in women with ovarian cancer. Several initiatives are ongoing towards a better characterization and immunomonitoring of human myeloid cells which may be of clinical benefit in future care [11]. In previous works, our and other research group demonstrated clinical relevance of myeloid cells in human ovarian cancer [2, 12–15]. However, clinical significance of tumour PD-L1 expression in OC patients is controversial. Whereas some reports indicate PD-L1 as a prognostic marker in ovarian cancer [16–18], no association between PD-L1 and prognosis in OC patients was found in other reports [19, 20]. Interestingly, recent publications have described elevated expression of PD-L1 on MDSCs and MO/MA [21] as well as elevated level of soluble PD-L1 (sPD-L1) in patients with some human cancers [8]. However, to the best of our knowledge, nobody hitherto compared in parallel the expression of PD-L1 on M-MDSCs, MO/MA, tumour cells (TC), tumour-infiltrating immune/inflammatory cells (IC) and the profile of sPD-L1 in different TMEs of ovarian cancer in the context of their clinical significance.

Here, we compared in parallel the expression of PD-L1 on M-MDSCs and MO/MA in the three different TMEs including blood, ascites and tumour tissue of ovarian cancer patients. In addition, we determined the profile of sPD-L1 in the both blood plasma and ascites fluid. Furthermore, we compared the expression of PD-L1 on TC and IC in the tumour tissue samples. The data were integrated with different clinicopathologic features of patients.

## Methods

### Study design and participant characteristics

A total number of 74 pretreatment women were enrollment to the study including 59 ovarian cancer patients and 15 healthy women. Blood (n = 43), ascites (n = 26) and tumour tissue (n = 29) samples from ovarian cancer patients were obtained 1 day before or during surgery at Ist Department of Oncologic Gynecology and Gynecology (Independent Public Clinical Hospital No. 1, Medical University of Lublin, Poland). Exclusion criteria for OC patients included (a) serious intercurrent acute/chronic illnesses; (b) presence of infections, allergic, autoimmune disorders; (c) presence of concurrent malignancy other than OC; (d) previous anticancer therapy prior to surgery; (e) intake of on immunosuppressants. While inclusion criteria were the following: (a) above 18 years old; (b) histologically confirmed diagnosis of OC. In some cases, the above requirements reduced the number of samples collected. Peripheral blood samples from Centre of Blood Donation and Blood Therapy were also collected from sex- and age-matched healthy donors with no history of malignancies or autoimmune diseases (n = 15) as a control group. In view of current inability to obtain normal/physiology ascites fluid and difficulty in collecting normal ovarian tissue, the distributions of ascites- and tumour-infiltrating M-MDSCs versus MO/MA, PD-L1^+^M-MDSC versus PD-L1^+^MO/MA and ascites sPD-L1 were compared in ovarian cancer patients. Kurman and Shih types of OC were analyzed as previously described [22]. Pre-existing clinical data, including age, FIGO stage, histopathologic grading, histological type, treatment history were collected from centralized database. Detailed characteristics of the patients are presented in Table 1. The study was approved by the Bioethical Committee of the Medical University of Lublin (no. KE-0254/299/2014). Prior to the study, each participant signed an informed consent and all experiments were performed out in accordance with ethical standards.

**Table 1:** Patients characteristics

Parameters	Ovarian cancer			Control
Material (type)	Blood	Ascites	Tumour tissue	Blood
Subjects, n	43	26	29	15
Age (years), med (min–max)	60 (20–86)	69 (20–86)	52 (20–80)	57 (34–64)
Stage, n (%)				NA
Early (I/II)	20 (46.5)	6 (23.1)	17 (58.6)	
Advanced (III/IV)	23 (53.5)	20 (76.9)	12 (41.4)	
Grade, n (%)				
GII	24 (55.8)	13 (50.0)	11 (37.9)	
GIII	19 (44.20	13 (50.0)	18 (62.1)	
Kurman and Shih’s type, n (%)				
I	28 (65.1)	13 (50.0)	21 (72.4)	
II	15 (34.9)	13 (50.0)	8 (27.6)	
Histology, n (%)				
Endometrioid	21 (48.8)	7 (26.9)	16 (55.2)	
Serous	18 (41.9)	13 (50.0)	9 (31.0)	
Mucinous	4 (9.3)	6 (23.1)	4 (13.8)	

*NA* not applicable

### Cells isolation

9 ml of peripheral blood were harvested into heparinized tubes (Sarstedt, Germany). Venous blood specimens were gathered before the operation procedure of OC patients. Fresh ascites fluid was collected aseptically and small pieces of neoplastically changed primary tumour tissue (~ 1 cm^3^) from nonmargin areas and without necrotic changes were obtained during the surgical resection. Blood and ascites specimens were centrifuged (1500 rpm/10 min) to obtain rendered cell-free fluids and the supernatants were immediately stored at − 80 °C for later analysis. OC patients’ blood plasma and ascites fluid were used immediately after defrosting and were not subjected to further freeze–thaw cycles. For isolation of tumour-infiltrating immune cells, freshly resected tumour tissue was minced with scissors into 2- to 4-mm, and placed into a gentleMACS C tube containing 5 ml of dissociation medium. Next, all tumour specimens were processed using Tumor Dissociation Kit (Miltenyi Biotec, Germany) to obtain single-cell suspension, following manufacturer’s instructions. The resulting cell suspension was filtered through 70-mm mesh filter (BD Biosciences, USA). Mononuclear cells (MCs) were obtained from blood, ascites and tumour tissue by gradient separation as we previously described in details [2] and were cryopreserved until use.

### Flow cytometric analysis

In this study we focused our attention on the cryoresistant myeloid cells (i.e. monocytic/macrophages and M-MDSCs) that can be easily recovered using density gradient centrifugation [23–25]. Mononuclear cells isolated from blood, ascites and tumour tissue were stained for flow cytometry analysis. The following combinations of monoclonal antibodies (mAbs) were used: PE-Cy7-conjugated anti-CD14 (Clone: M5E2, Catalog No. 557742), PerCP-Cy5.5-conjugated anti-HLA-DR (Clone: G46-6, Catalog No. 560652), and PE-conjugated anti-PD-L1 (CD274/B7-H1 Clone: MIH1, Catalog No. 557924) (all from BD Biosciences, USA). Briefly, 100 μl of cells were incubated in the dark with conjugated anti-human mAbs for 30 min at room temperature. Nonspecific staining was prevented by blocking Fc receptors using Fc receptor blocking agent (Miltenyi Biotec, Germany). After staining, the cells were evaluated using BD FACSCanto flow cytometer (BD Biosciences, USA). Flow data were gathered in FACS DIVA software (BD Biosciences, USA) and the data were analized using FCS Express 6 Flow Cytometry (De Novo Software, USA). The compensation control was performed with BD CompBeads set (BD Biosciences, USA) using the manufacturer’s instructions. Fluorescence minus one (FMO) controls were used as negative controls. The level of PD-L1-expressing cells was calculated as the percentage of the total respective subset (i.e. HLA-DR^−/low^CD14^+^ M-MDSCs and HLA-DR^+^CD14^+^ MO/MA).

### Analysis of soluble PD-L1

The concentrations of sPD-L1 were examined in the same available samples which were analyzed by flow cytometry. Blood plasma (n = 39) and ascites fluid (n = 22) were analyzed using ELISA (EIAab, China) according to the manufacturer’s protocol. All measurements were determined on the same day to avoid inter-assay variations. Plate absorbance was quantified using a ELX-800 Universal Microplate Reader (Bio-Tek, USA). Concentration of sPD-L1 (pg/ml) was calculated by interpolation from a standard curve. Gen5™ software (Bio-Tek, Instruments, USA) was used for the acquisition and data analysis.

### RNA extraction and quantitative reverse transcription PCR (qRT-PCR)

For qPCR analysis of mononuclear cells from paired patients’ blood, ascites and tumour tissue samples (n = 10), the following TaqMan^®^ probes were used: glyceraldehyde 3-phosphate dehydrogenase (GAPDH; Hs03929097_g1) and PD-L1 (Hs00204257_m1) (Thermo Fisher Scientific, USA). Total RNA of the blood, ascites and tumour tissue MCs was extracted from OC patients with AllPrep DNA/RNA/Protein Mini Kit (Qiagen, Germany) according to the manufacturer’s protocol. cDNA was synthetized with High Capacity RNA-to-cDNA Kit (Life Technologies, USA). qPCR was done using CFX96 Touch™ Real-Time PCR Detection System (Bio-Rad, CA, USA). Data were analyzed in CFX™ Manager Software (Bio-Rad, USA). Reactions for all samples were run in triplicate and 1uL cDNA sample was used for reaction. After 5 min of initial denaturation at 95 °C, cDNA was amplified in 50–60 cycles (denaturation for 15 s at 95 °C and annealing for 60 s at 60 °C). The expression of PD-L1 mRNA was normalized to GAPDH gene and the data were examined using 2^−ΔΔCT^ calculation.

### Immunohistochemistry analysis of PD-L1 on tumour cells and tumour-infiltrating immune/inflammatory cells

Microscopic preparations were performed from the paraffin-embedded blocks which contain representative samples of tissue collected from ovarian cancer. The immunohistochemistry (IHC) was performed on 4 μm tissue sections as we previously described [26]. Briefly, primary antibody against the PD-L1 (PDL1/2746; ab238697, Abcam, USA) was used. Staining procedures weres performed according to primary antibody manufacturer’s recommendations. For tumour cells the proportion of PD-L1-positive cells was evaluated as the percentage of total tumour cells. For tumour-infiltrating immune/inflammatory cells, the percentage of PD-L1-positive tumour-infiltrating immune cells occupying the tumour was analyzed [27]. The reactions were evaluated with the use of light microscope Olympus BX45 (Olympus, Japan). Analysis was prepared independently by two experienced observers, blinded to clinical outcome, one being a board-certified pathologist. In case of discrepancies, slides were re-evaluated to reach consensus. To evaluate the PD-L1 expression in both TC and IC populations, scoring system, combining quantity (number of PD-L1 positive cells) and quality (intensity of colour reaction) features, was used. Cells from both populations were categorized by staining intensity (negative expression/staining intensity = 0; low expression/staining intensity = 1, medium expression/staining intensity = 2 and high expression/staining intensity = 3). Cells in each cathegory were counted and expressed as percent of all visible cells from particular population (TC or IC). The value of expression score was calculated by following formula:

$$\frac{{\left( {{\text{\% of low intensity cells }} \times 1} \right) + \left( {{\text{\% of medium intensity cells }} \times 2} \right) + \left( {{\text{\% of high intensity}}\;{\text{cells }} \times 3} \right)}}{{100{\text{\% }}}}$$

The results of described calculation are real numbers and were used for statistical analysis.

### In silico database analysis

For in silico analysis Kaplan–Meier plotter database was used to further analyze the prognostic relevance of PD-L1 (CD274) mRNA expression using gene chip data in OC [28]. Only optimal probe set (JetSet best probe set) for PD-L1 was used. In this Kaplan–Meier plotter database, information about PD-L1 mRNA expression and OS in OC patients were obtained from large independent cohort (n = 655) available from all datasets together and from seven datasets separately including GSE18520 (n = 53), GSE19829 (n = 28), GSE26193 (n = 107), GSE27651 (n = 39), GSE30161 (n = 50), GSE63885 (n = 25) and GSE9891 (n = 285).

### Statistical analysis

The differences between healthy women and OC patients were assessed by Student’s t test using 2-tailed Mann–Whitney U test. Correlations were investigated using Spearman’s rank correlation analysis. Data are expressed as medians (range). p values less than 0.05 were considered statistically significant. OS was evaluated and performed using log-rank test. Kaplan–Meier curves were demonstrated. All analyses were carried out by using the GraphPad Prism 5 (GraphPad Software, USA). For futher validation of prognostic relevance of PD-L1 mRNA, the Kaplan–Meier plotter tool was used (http://kmplot.com/analysis/) [28].

## Results

### Monocytes/macrophages are increased relative to MDSCs in the blood, ascites and tumour tissue of ovarian cancer patients

**Fig. 1:**
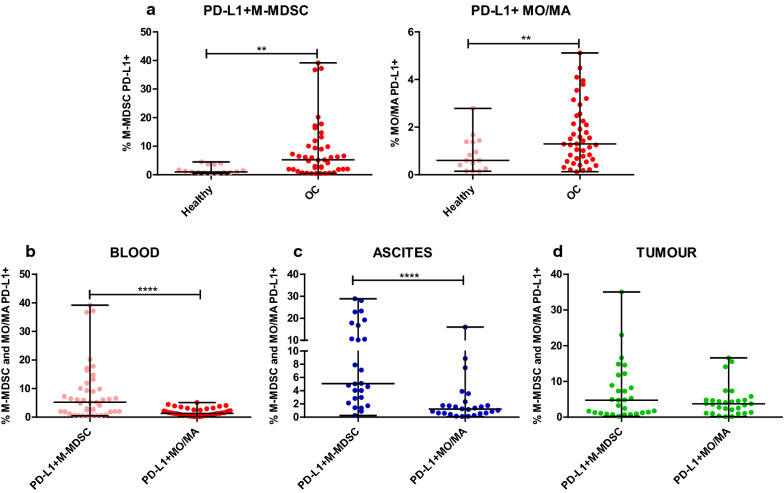
Evaluation of HLA-DR^−/low^CD14^+^ monocytic myeloid-derived suppressor cells (M-MDSCs) and HLA-DR^+^CD14^+^ monocytes/macrophages (MO/MA). Simultaneous analysis of myeloid cell populations in the **a**, **b** blood, **c** ascites and **d** tumour tissue of patients with ovarian cancer. Mononuclear cells (MCs) obtained from the blood (n = 43), ascites (n = 26), and tumour tissue (n = 29) of ovarian cancer patients and from the blood of healthy women (n = 15) were analyzed using flow cytometry. The levels of M-MDSCs and MO/MA are presented as the percentage of MCs. The horizontal lines are the median values and the whiskers indicate the minimum and maximum values. Each point corresponds to an individual patient. *p < 0.05; ***p < 0.001; ****p < 0.0001

Firstly, we examined the frequency of two populations of myeloid cells. i.e. HLA-DR^−/low^CD14^+^ M-MDSCs and HLA-DR^+^CD14^+^ MO/MA among mononuclear cells (MCs) isolated from the three TMEs including blood, ascites and tumour tissue. For M-MDSCs and MO/MA characterization, we used gating strategy which defines human M-MDSC as HLA-DR^−/low^CD14^+^ and MO/MA as HLA-DR^+^CD14^+^. The frequency of myeloid cells are presented as the percentage of MCs. Next, we identified the expression profiles of PD-L1 on these myeloid cell subsets. The expression levels of PD-L1 were presented as the percentage of the total respective cell subsets (i.e. M-MDSC and MO/MA) (Additional file 1: Fig. S1). It has been demonstrated that the level of different myeloid cell populations was alerted in ovarian cancer patients [2, 5]. However, parallel evaluation of M-MDSCs and MO/MA in the three TMEs of ovarian cancer patients is unknown. We selected pretreatment OC patients who had not received any therapy at the first diagnosis to exclude the effect of any treatment on blood, ascites and tumour tissue immune cells frequencies. Blood samples were also obtained from healthy women (n = 15). Due to the current inability to obtain ascites fluid and ovarian tissue from healthy individuals, the distribution of examined immunosuppressive factors were compared in ovarian cancer patients. The percentage of M-MDSCs and MO/MA was higher in the blood of patients compared to healthy women (p < 0.001 and p < 0.05, respectively, Fig. 1a). Furthermore, we observed higher accumulation of blood-circulating MO/MA versus M-MDSC in the ovarian cancer patients (p < 0.0001, Fig. 1b). Similarly, we showed higher level of MO-MA compared to M-MDSCs in the ascites (p < 0.0001, Fig. 1c) and tumour tissue (p < 0.0001, Fig. 1d) of ovarian cancer patients. Next, we asked the question if differences exist between distribution of examined myeloid cell populations in the three different TMEs (blood, ascites and tumour tissue). We observed no significant disparity in the distribution of both M-MDSC and MO/MA in the all three examined TMEs (p > 0.05) (Additional file 2: Fig. S2a). Summarizing, we demonstrated higher abundance of monocytes/macrophages compared with M-MDSCs in all three TMEs of ovarian cancer patients.

### Accumulation of M-MDSCs and monocytes/macrophages in the three TMEs is clinicopathologic-independent

**Fig. 2:**
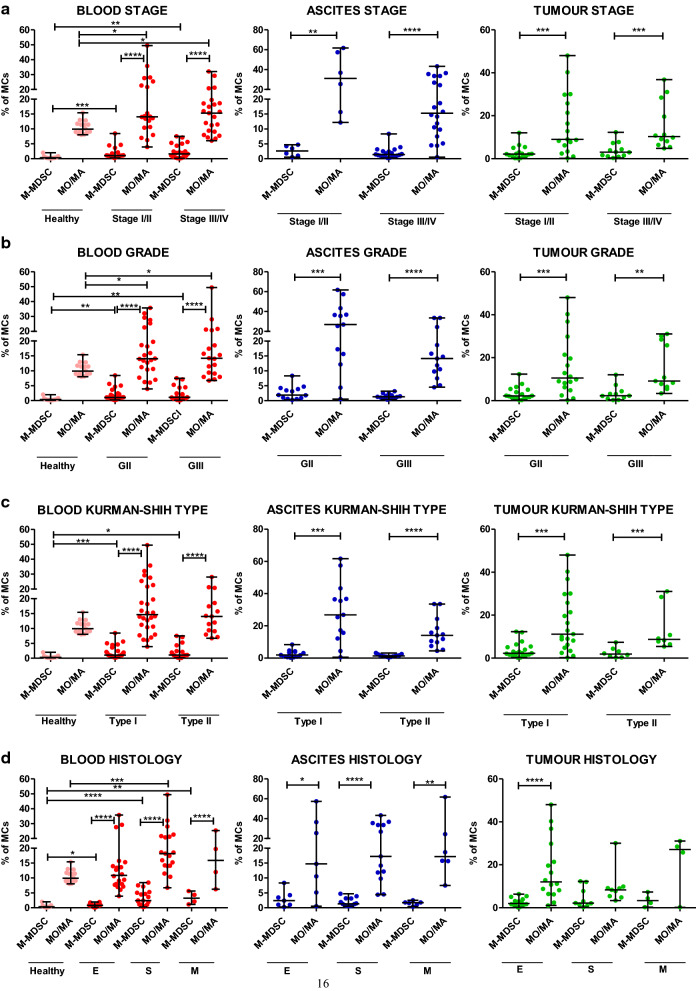
Parallel evaluation of HLA-DR^−/low^CD14^+^ monocytic myeloid-derived suppressor cells (M-MDSCs) and HLA-DR^+^CD14^+^ monocytes/macrophages (MO/MA) in ovarian cancer patients with different clinicopathologic characteristics. **a**–**d** Samples from the blood (n = 43), ascites (n = 26) and tumour tissue (n = 29) of patients with different stage, grade, Kurman–Shih types and histology types of OC. Blood samples from healthy women were also examined (n = 15). The frequency of M-MDSCs and MO/MA is shown as the percentage of mononuclear cells (MCs). The horizontal lines represent the median values and the whiskers indicate the minimum and maximum values. Each point represents an individual patient. *p < 0.05; **p < 0.01; ***p < 0.001; ****p < 0.0001

Next, in order to assess clinical relevance of M-MDSCs and MO/MA, we determined their association with the patients’ clinical characteristics. The abundance of blood-circulating M-MDSCs and MO/MA was higher in low I/II (p < 0.001 and p < 0.05, respectively) and advanced III/IV (p < 0.01 and p < 0.05, respectively) stages (Fig. 2a), grade II (p < 0.01 and p < 0.05, respectively) and III (p < 0.01 and p < 0.05, respectively) tumours compared with healthy women (Fig. 2b). Similarly, a higher level of blood-circulating M-MDSCs but not MO/MA was observed in both Kurman–Shih types of OC (p < 0.001 and p < 0.05, respectively) versus healthy women (Fig. 2c). When comparing ovarian cancer patients with different histological types, we found a significantly elevated frequency of blood-circulating M-MDSCs in all three types of tumours including endometrioid (p < 0.05), serous (p < 0.0001) and mucinous OC (p < 0.01) compared to control. In contrast, a higher level of blood-circulating MO/MA was observed only in serous OC (p < 0.001) (Fig. 2d). We demonstrated a higher level of blood-circulating MO/MA versus M-MDSCs in all stages, grades, Kurman–Shih types and histology types of OC (each p < 0.0001, Fig. 2a–d). Similarly, we showed significantly higher frequency of ascites-infiltrating MO/MA compared with M-MDSCs in two stages (p < 0.01 and p < 0.0001, respectively, Fig. 2a) grades (p < 0.001 and p < 0.0001, respectively, Fig. 2b), Kurman Shih types (p < 0.01 and p < 0.0001, respectively, Fig. 2c) and in the all three histology types of OC (p < 0.05, p < 0.0001 and p < 0.01, respectively, Fig. 2d). As we expected, higher abundance of MO/MA versus M-MDSCs was also observed in tumour tissue in two stages (both p < 0.001, Fig. 2a) grades (p < 0.001 and p < 0.01, respectively, Fig. 2b) and Kurman Shih types of OC (both p < 0.001 Fig. 2c). In contrast, significantly higher level of tumour-infiltrating MO/MA compared with M-MDSCs was observed only in endometrioid type of OC (p < 0.0001, Fig. 2d). Interestingly, clinical analysis revealed no other significant disparity in the distribution of M-MDSCs and MO/MA in the blood, ascites and tumour tissue in different stages, grades, Kurman–Shih types and histology types of OC (p > 0.05, Fig. 2a–d). Summarizing, our results demonstrated clinicopathologic-independent accumulation of both M-MDSCs and MO/MA in the all three examined TMEs of ovarian cancer patients.

### PD-L1 expression on M-MDSCs compared with monocytes/macrophages is increased in the blood and ascites of ovarian cancer patients

**Fig. 3:**
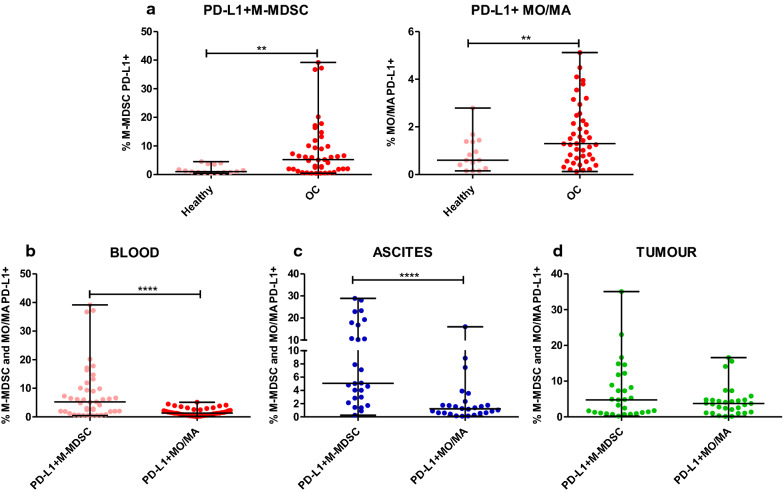
Expression profile of programmed death-ligand 1 (PD-L1). Simultaneusly analysis of PD-L1 on HLA-DR^−/low^CD14^+^ monocytic myeloid-derived suppressor cells (M-MDSCs) and HLA-DR^+^CD14^+^ monocytes/macrophages (MO/MA) in the **a**, **b** blood, **c** ascites and **d** tumour tissue of patients with ovarian cancer. Mononuclear cells (MCs) obtained from the blood (n = 43), ascites fluid (n = 26), and tumour tissue (n = 29) of ovarian cancer patients and blood of healthy women (n = 15) were analyzed by flow cytometry. The frequencies of PD-L1^+^ cells are presented as the percentage of the total respective subsets (HLA-DR^−/low^CD14^+^ M-MDSCs and HLA-DR^+^CD14^+^ MO/MA). The horizontal lines are the median values and the whiskers indicate the minimum and maximum values. Each point corresponds to an individual patient. **p < 0.01; ****p < 0.0001

Guided by the reported immunosuppressive activity of MDSCs and monocytes/macrophages in OC [5], we prepared a comparative analysis of its expression in the three TMEs. We observed higher accumulation of blood-circulating PD-L1^+^M-MDSCs and PD-L1^+^MO/MA in the patients versus healthy women (both p < 0.01, Fig. 3a). Intriguingly, although we found the higher percentage of MO/MA versus M-MDSCs in the ovarian cancer, the expression level of PD-L1 on M-MDSCs was higher compared to MO/MA in the blood of OC patients (p < 0.0001, Fig. 3b). Similarly, we revealed higher accumulation of PD-L1^+^M-MDSCs compared to PD-L1^+^MO/MA in the ascites (p < 0.0001, Fig. 3c) but not in the tumour tissue samples (p > 0.05, Fig. 3d). When we compared distribution of two PD-L1^+^ myeloid cell populations in the paired blood, ascites and tumour tissue samples from ovarian cancer patients, we observed no significant disparity (p > 0.05, Additional file 2: Fig. S2b). In order to characterize more deeply PD-L1 expression, we expanded our analysis by gene expression analysis of PD-L1 on the mononuclear cells isolated from the same paired patients’ blood, ascites and tumour tissue samples. Similarly to cytometric results, we found similar levels of PD-L1 mRNA in the all three examined TMEs (Additional file 2: Fig. S2c). Summarizing, we revealed higher abundance of PD-L1-expressing M-MDSCs versus PD-L1-expressing MO/MA in the blood and ascites of ovarian cancer patients.

### Accumulation of PD-L1-expressing M-MDSCs and monocytes/macrophages in the three TMEs is similar in ovarian cancer patients with different clinicopathologic characteristics

**Fig. 4:**
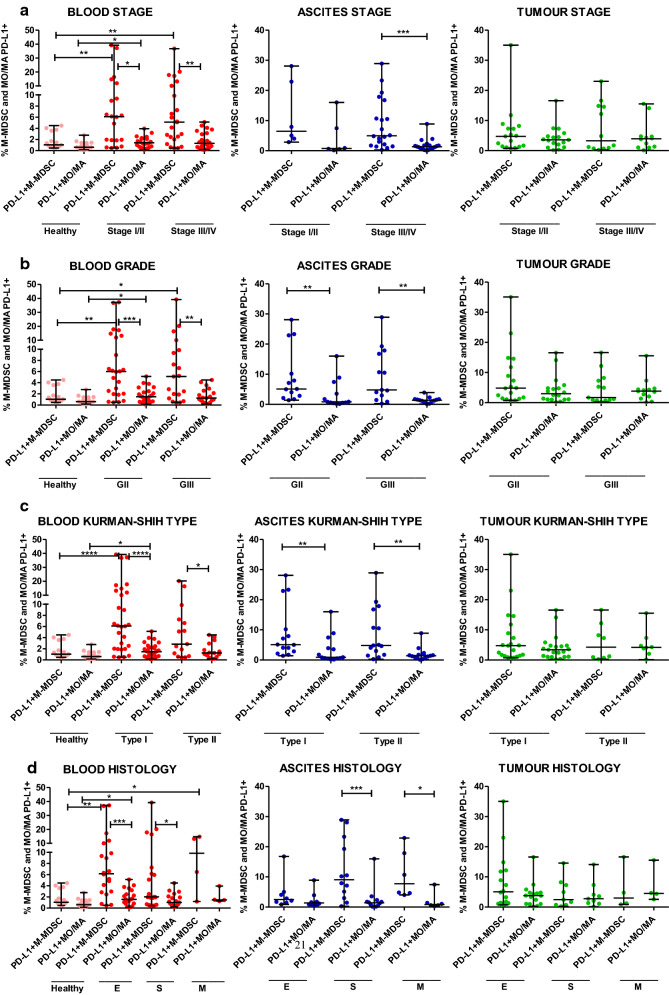
Parallel evaluation of the expression profile of programmed death-ligand 1 (PD-L1) on HLA-DR^−/low^CD14^+^ monocytic myeloid-derived suppressor cells (M-MDSCs) and HLA-DR^+^CD14^+^ monocytes/macrophages (MO/MA) in ovarian cancer patients with different clinicopathologic characteristics. **a**–**d** The blood (n = 43), ascites (n = 26) and tumour tissue (n = 29) specimens of patients with different stage, grade, Kurman–Shih type and histology were analyzed. The blood samples from healthy women were also examined (n = 15). The frequencies of PD-L1^+^cells are presented as the percentage of the total respective subsets (HLA-DR^−/low^CD14^+^ M-MDSCs and HLA-DR^+^CD14^+^ MO/MA). The horizontal lines are the median values and the whiskers indicate the minimum and maximum values. Each point represents an individual patient. *p < 0.05; **p < 0.01; ***p < 0.001; ****p < 0.0001

In the next step, we examined the clinical relevance of PD-L1^+^M-MDSC and PD-L1^+^MO/MA. The abundance of blood-circulating PD-L1^+^M-MDSCs was higher in early I/II and advanced III/IV stages (both p < 0.01, Fig. 4a), grades II and III (p < 0.01 and p < 0.05, respectively, Fig. 4b), Kurman–Shih type I (p < 0.0001, Fig. 4c), endometrioid and mucinous (p < 0.01 and p < 0.05, respectively, Fig. 4d) OC compared with healthy women. In contrast, the level of blood-circulating PD-L1^+^M-MDSCs was higher only in early stage (p < 0.05, Fig. 4a), grade II (p < 0.05, Fig. 4b), type I (p < 0.05, Fig. 4c), and endometrioid (p < 0.05, Fig. 4d) OC versus control. Higher abundance of blood-circulating PD-L1^+^M-MDSCs versus PD-L1^+^MO/MA was observed in both stages (p < 0.05 and p < 0.01, respectively, Fig. 4a), grades (p < 0.001 and p < 0.01, respectively, Fig. 4b), Kurman Shih types (p < 0.0001 and p < 0.05, respectively, Fig. 4c), endometrioid and serous (p < 0.001 and p < 0.05, respectively, Fig. 4d) OC compared with healthy women. Interestingly, the clinicopathologic analysis of cancer patients revealed a higher level of ascites- but not tumour-infiltrarting PD-L1^+^M-MDSCs versus PD-L1^+^MO/MA in advanced stage (p < 0.001, Fig. 4a), grades II and III (both p < 0.01, Fig. 4b), type I and II (both p < 0.01, Fig. 4c) as well as both serous (p < 0.001) and mucinous (p < 0.05) OC (Fig. 4d). We showed no other significant disparity in the distribution of PD-L1^+^myeloid cells in different clinicopathologic features of ovarian cancer patients (Fig. 4a–d). Similarly to the distribution of M-MDSCs and MO/MA, our results revealed independent to various clinical characteristics of OC patients (i.e. stage, grade, Kurman–Shih type, histology) expression profile of the PD-L1 on M-MDSCs and MO/MA.

### Ovarian cancer patients have increased levels of sPD-L1 which correlate with PD-L1^+^ M-MDSCs and PD-L1^+^ MO/MA in the blood

**Fig. 5:**
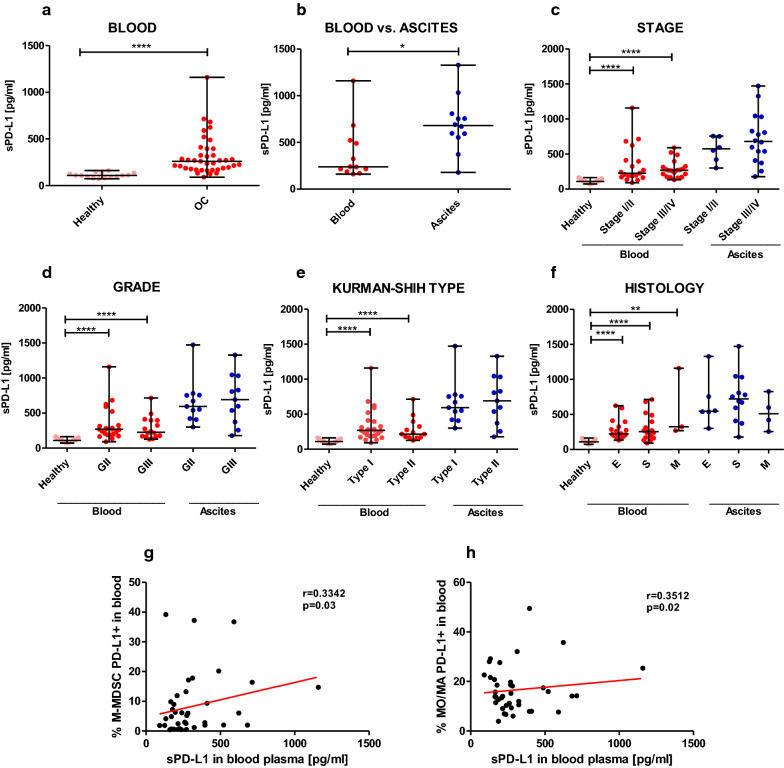
Production of soluble programmed death ligand 1 (sPD-L1) in the blood plasma (n = 39) and ascites fluid (n = 22) of ovarian cancer patients. **a** sPD-L1 level in the blood plasma of ovarian cancer patients and healthy women. **b** Comparative analysis of sPD-L1 level in the paired blood plasma and ascites fluid samples of ovarian cancer patients (n = 11). **c**–**f** The level of sPD-L1 in the blood plasma and ascites fluid samples of ovarian cancer patients with different stage, grade, Kurman–Shih type and histology. **g** The correlation between the abundance of PD-L1^+^M-MDSCs and sPD-L1 in the blood plasma from ovarian cancer patients (n = 39). **h** The correlation between the abundance of PD-L1^+^MO/MA and sPD-L1 in the blood plasma from ovarian cancer patients (n = 39). sPD-L1 concentrations were examined using enzyme-linked immunosorbent assay (ELISA). Horizontal lines indicate the median and the whiskers indicate the minimum and maximum values. Each point represents an individual patient. *p < 0.05; **p < 0.01; ****p < 0.0001

Guided by the reported few studies on clinical relevance of sPD-L1 in human malignancies [8] we assessed in parallel its level in the patients’ blood plasma and ascites fluid as well as blood plasma in healthy women and integrated these data with clinical features. We used the same available blood and ascites specimens which were analyzed by flow cytometry. ELISA was used to establish sPD-L1 concentration, as this test is much faster than flow cytometry and sPD-L1 can be used as a surrogate marker of immune cells (i.e. M-MDSCs and monocytes/macrophages) surface expression of PD-L1. We found a higher level of blood plasma sPD-L1 in patients compared with healthy women (med. 260 vs. 109 pg/ml, respectively, p < 0.0001, Fig. 5a). When we comparing paired samples of blood and ascites from ovarian cancer patients, we showed higher concentration of ascites fluid versus blood plasma sPD-L1 (med. 680 vs. 238.5 pg/ml, respectively, p < 0.05, Fig. 5b). Interestingly, we observed higher concentrations of blood plasma sPD-L1 in both stages, grades and Kurman–Shih types of OC patients compared with healthy women (each p < 0.0001, Fig. 5c–e, respectively). Furthermore, we found higher concentrations of blood plasma sPD-L1 in endometrioid, serous and mucinous type of OC versus control group (p < 0.0001, p < 0.0001 and p < 0.01, respectively, Fig. 5f). We observed no significant disparity between the distribution of blood and ascites sPD-L1 and the different clinical features of OC patients (p > 0.05, Fig. 5c–f). Similarly to the distribution of myeloid cells and PD-L1-positive myeloid cells our results revealed clinicopathologic-independent concentrations of sPD-L1 in the disease.

Next, we asked if the relationship between PD-L1-expressing MDSCs/MO/MA and sPD-L1 in the blood and ascites of OC patients exists. We could distinguish significantly positive correlations with the concentrations of sPD-L1 and the levels of expression of PD-L1 on both M-MDSCs (p = 0.03, Fig. 5g) and MO/MA (p = 0.02, Fig. 5h) in the blood but not in the ascites (p > 0.05, data not shown) of ovarian cancer patients.

### Expression of PD-L1 on inflammatory/immune cells is higher relative to expression of PD-L1 on tumour cells

**Fig. 6:**
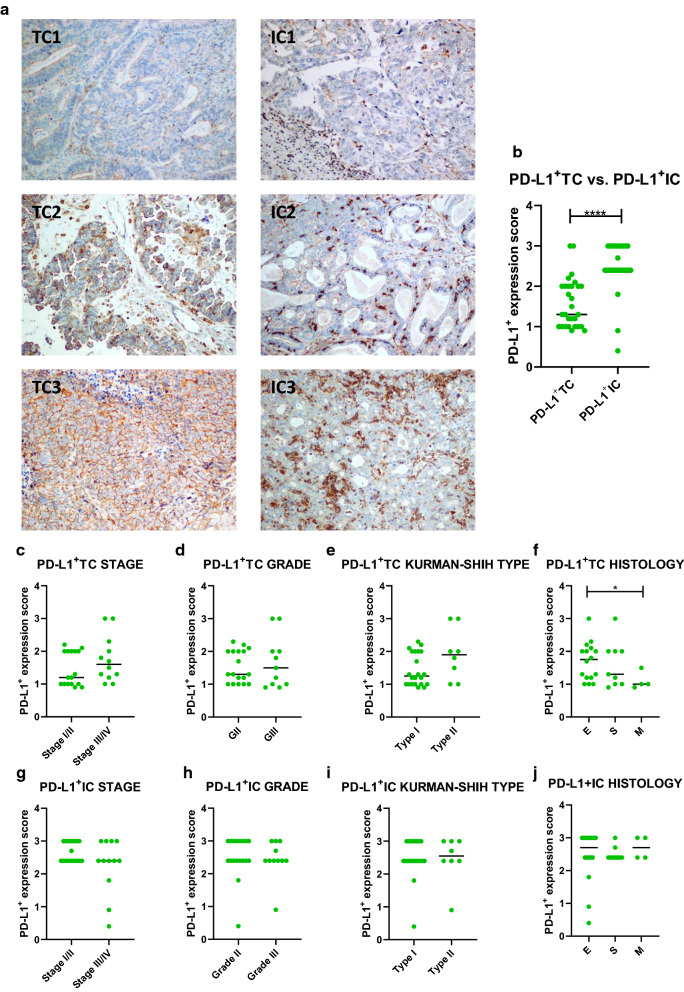
PD-L1 prevalence in tumour tissue of ovarian cancer patients. Immunohistochemistry (IHC) tissue staining of PD-L1 on tumour cells (TC) and tumour-infiltrating inflammatory/immune cells (IC) (n = 29). **a** Representative images (×10 magnification) showing PD-L1 IHC on tumour cells (TC) and tumour-infiltrating immune/inflammatory cells (IC); TC/IC1 (low expression); TC/IC2 (medium expression); TC/IC3 (high expression). **b** PD-L1^+^TC and PD-L1^+^IC expression in tissue samples. PD-L1^+^TC in OC tissue based on **c** stage, **d** grade, **e** Kurman–Shih type and **f** histologic type of tumours. PD-L1^+^ IC in tissue samples based on **g** stage, **h** grade, **i** Kurman–Shih type and **j** histologic type of tumours

Tissue staining was prepared in the same patients cohort which was used for cytometric analysis. Tumour cell-specific and immune cell-specific PD-L1 expression could be assessed in 29 (100%) cases. Different percentage of cells (0–100%) showed different expression/staining intensity (0–3) of PD-L1. To calculate the score of PD-L1 expression for TC/IC both the  % of cells and expression level/staining intensity were used. Sample IHC images are shown in Fig. 6a The score of PD-L1-positive tumour-infiltrating immune cells was almost two fold higher than PD-L1-positive tumour cells (med. 2.4 versus 1.3, respectively p < 0.0001, Fig. 6b). PD-L1 expression analysis on both TC and IC based on patients’ clinical characteristics demonstrated no significant differences in the stages, grades, Kurman–Shih types of OC (p > 0.05, Fig. 6c–e, g–i). In contrast, we observed higher level of PD-L1^+^TC but not PD-L1^+^IC in the endometrioid tumours versus mucinous type of OC (p < 0.05 and p > 0.05, respectively, Fig. 6f, j). We observed no correlation between PD-L1-expressing TC/IC and analyzed factors (i.e. M-MDSCs, MO/MA, PD-L1^+^M-MDSC, PD-L1^+^MO/MA and sPD-L1, each p > 0.05, data not shown).

Finally, we asked about prognostic effect of PD-L1 and sPD-L1. We observed no significant association between level of PD-L1^+^M-MDSC, PD-L1^+^MO/MA, sPD-L1, PD-L1^+^TC, PD-L1^+^IC and OS of patients (each p > 0.05, Additional file 3: Fig. S3a–j). The Kaplan–Meier plotter database further demonstrated that CD274 (PD-L1) mRNA expression was not associated with OS in 655 patients with ovarian cancer (all datasets analysis) and in seven datasets which were analyzed separately, including GSE18520 (n = 53), GSE19829 (n = 28), GSE26193 (n = 107), GSE27651 (n = 39), GSE30161 (n = 50), GSE63885 (n = 25) and GSE9891 (n = 285) (each p > 0.05, Additional file 4: Fig. S4a–h).

## Discussion

To the best of our knowledge, this is the first report demonstrated positive association between sPD-L1 and PD-L1-postive myeloid cells including M-MDSCs and monocytes/macrophages in the blood of human cancers. Although the importance of myeloid cells in ovarian cancer have been reported by our and other research group [2–5] no comparative evaluation of PD-L1 expression on M-MDSCs, monocytes/macrophages, tumour cells and inflammatory/immune cells is available to date. The data of sPD-L1 in blood of ovarian cancer have been reported [29, 30], however no study has been published yet regarding conjointly evaluation of this soluble factor in the blood plasma and ascites fluid. Herein, we comparatively evaluate of PD-L1-expressing M-MDSCs and MO/MA in the three different TMEs including blood, ascites and tumour tissue as well as investigate sPD-L1 profile in the blood plasma and ascites fluid of ovarian cancer patients. Furthermore, we compared the expression of PD-L1 on tumour cells and inflammatory/immune cells in the tumour tissue samples. We integrated data with clinicopathologic features of patients.

In this study, we showed that both blood-circulating M-MDSCs and MO/MA were increased in OC patients compared with controls. It has been reported that CD14^+^HLA-DR^−/low^ MDSCs in the blood are increased in patients with ovarian cancer [14], and we obtained similar results in this study. Furthermore, our study showed higher accumulation of blood-circulating, ascites- and tumour-infiltrating MO/MA compared to M-MDSCs in OC patients. The results from our and other research groups demonstrated that M-MDSC represents a very small fraction of mononuclear cells [2, 7]. However, in non-small cell lung cancer (NSCLC), the data revealed a higher level of blood-circulating M-MDSCs than monocytes/macrophages [31]. In the present study, we found no significant disparity in the distribution of MDSC and MO/MA in the three examined TMEs including blood, ascites and tumour tissue. To the contrary, in another study, monocytes/macrophages were significantly more abundant in the lymph nodes and tumour tissues than in blood from NSCLC patients. Besides, the percentage of M-MDSCs was significantly higher in the blood than the lymph nodes and tumour tissue of these patients [31]. Our data indicate that high accumulation of both M-MDSCs and monocytes/macrophages in OC can be TMEs-independent.

Given the results of our and previous research groups regarding immunosuppressive activity of myeloid cells and their clinical relevance [2, 5, 32], we next compared expression of PD-L1 on M-MDSC and monocytes/macrophages in the three TMEs of ovarian cancer patients with different clinicopathologic features. PD-L1 is a key mediator of immunosuppressive activity of myeloid cells by interaction with the receptor of PD-1 on T cells [6]. We showed that both blood-circulating PD-L1^+^M-MDSC and PD-L1^+^MO/MA were increased in the patients versus controls, which demonstrate upregulated immunosuppressive activity of these cells in OC blood. In accordance with our study, other group reported that the frequency of circulating CD33^+^HLA-DR^low/−^CD11b^+^CD14^+^PD-L1^+^MDSCs increased in hepatocellular carcinoma (HCC) patients versus controls. In contrast to our study, the frequency of PD-L1^+^MDSCs increased with the progression of HCC [33]. Interestingly, although the percentage of MO/MA was higher compared to M-MDSC in patients, the expression level of PD-L1 was higher on M-MDSC versus MO/MA in the blood and ascites of cancer patients. Our data indicate that M-MDSCs can be more immunosuppressive cells than monocytes/macrophages. Similarly, HLA-DR^low/−^CD14^+^ cells from esophageal squamous cell carcinoma patients expressed elevated PD-L1 comparing to controls [34]. Besides, PD-L1-highly expressing M-MDSCs were also detected in squamous cell carcinoma of the head and neck [35]. Intriguingly, although we observed higher level of PD-L1^+^M-MDSC versus PD-L1^+^MO/MA in the blood and ascites of cancer patients, we do not see such disparity in the tumour tissue. One explanation can be that the expression of PD-L1^+^myeloid cells in tumour sample is heterogeneous and small tissue specimens might not be representative for whole tumour. It is widely known that ovarian cancer tumours characterize both inter- and intra-tumoural heterogeneity [36], thus the routine clinical tumour biopsy possess limitations due to the need to gather representative biopsies from the entire tumour. However, assessing the immune factors in the circulation (i.e. liquid biopsy) of OC patients can better capture this heterogeneity and may represent a feasible approach in personalized oncology. We observed no significant disparity in the expression of PD-L1 on both MDSC and MO/MA in the three examined TMEs in OC. This indicates that high expression of PD-L1 on MDSCs/monocytes/macrophages is characteristic of all three examined TMEs of ovarian cancer. Furthermore, the accumulation of both PD-L1^+^M-MDSCs and PD-L1^+^MO/MA is clinicopathologic-independent in ovarian malignancy. In accordance with our study, Yamauchi et al. showed that frequencies of circulating PD-L1^+^M-MDSCs were higher in the NSCLC patients than in the controls. However, they failed to observe any significant changes in the frequencies of PD-L1^+^M-MDSCs during tumour progression [21], which fits to our finding of no significant changes in the accumulation of PD-L1^+^M-MDSCs in OC patients with different clinical features.

In the light of previous findings about the profile of sPD-L1 and its clinical relevance in cancer patients [8, 29, 30], we hypothesized that the levels of sPD-L1 might be of clinical value in patients with OC. Thus, we performed an analysis of both blood plasma and ascites fluid levels of sPD-L1 and integrated these data with clinical characteristics of patients. Interestingly, we showed highly elevated levels of blood plasma sPD-L1 in patients compared with controls as well as higher accumulation of sPD-L1 in the ascites fluid versus blood plasma. It is in accordance with reports which presented a higher level of serum sPD-L1 in multiple myeloma, lung cancer and ovarian cancer patients [29, 30, 37, 38]. Similarly, Zhang et al. reported that the average levels of sPD-L1 in patients with advanced NSCLC and controls were significantly different [39]. The reason for greater abundance of sPD-L1 in ascites fluid compared with blood plasma samples of OC patients is not well understood. Recent study demonstrated that the secretion of exosomal sPD-L1 correlated with tumour size, suppressed CD8 T cells and facilitated tumour growth [40]. Our data led us to speculate that highly elevated sPD-L1 production in the ascites, as a typical habitat for OC lesions, plays an important role in the peritoneal dissemination and development of malignancy as well as creates strongly immunosuppressive milieu [41]. Therefore, high concentrations of sPD-L1 in the peritoneal cavity might be one of the relevant factors for the poor efficacy of immunotherapy in ovarian cancer. Although immunotherapy has not been yet approved for the standard treatment of OC [42], during the following years, several thousand women with malignancy will be included in the clinical trials to assess immunotherapy efficacy. We observed no significant differences in the distribution of blood and ascites sPD-L1 in the relation to the disease status. However, we showed that the levels of sPD-L1 in the blood plasma were higher in the OC patients regardless of the clinicopathologic characteristics compared with control group. These data led us to surmise that high sPD-L1 production in the blood and peritoneal cavity may be a characteristic feature of OC, independently of the status of the disease. Similarly, another study reported that sPD-L1 levels in NSCLC patients were not correlated with any clinicopathologic features except for tumour size [43]. However, another study showed significantly higher levels of sPD-L1 in the plasma of patients with OC compared with patients with benign tumours [44]. These results indicated sPD-L1 as a discriminatory marker of benign and malignant ovarian lesions rather than particular types of ovarian cancer.

To assess the distribution of PD-L1 on tumour tissue of ovarian cancer patients, we compared PD-L1 expression on both tumour cells and tumour-infiltrating immune/inflammatory cells. To date, no comprehensive analysis of both IC and TC have focused on OC. Indeed, in the majority of related analysis, PD-L1 expression in tumor sections was evaluated without discrimination between TC and IC. Interestingly, we observed almost two fold higher level of PD-L1 on immune cells versus tumour cells. This is in line with previous report which demonstrated that PD-L1-positive tumour infiltrating IC are more common than PD-L1-positive TC in other human cancers [27]. Similar to PD-L1-expressing myeloid cells and sPD-L1, we found clinicopathologic-independent accumulation of both PD-L1^+^TC and PD-L1^+^IC, except higher expression of PD-L1^+^TC but not PD-L1^+^IC in the endometrioid versus mucinous type of tumours, which is in accordance with meta-analysis of Wang et al. where showed that PD-L1 protein expression was not associated with clinical features of OC (i.e. grade, stage or lymph node status) [45].

Recently, it has been shown that sPD-L1 can be detected in the sera of patients, which correlates with amount of PD-L1 expressing cells [46]. An important finding in our study is that blood plasma sPD-L1 correlates with blood-circulating PD-L1^+^MDSCs and PD-L1^+^MO/MA but not with PD-L1^+^TC nor PD-L1^+^IC in tumour tissue. It is worth noting that sPD-L1 is detectable in supernatants from PD-L1^+^ cell lines rather than in those from PD-L1^−^ cell lines [33], thereby indicating that PD-L1 expressed on the cell surface might be a source of sPD-L1. Although researchers have found that both immune and tumour cells can be sources of sPD-L1 [47, 48], no significant correlation of sPD-L1 with tumour PD-L1 expression was determined in patients with HCC, pancreatic cancer, diffuse large B-cell lymphomas and renal cell carcinomas [49–52], which is in line with our IHC results. Among the immune cells, sPD-L1 has been detected in cultures of monocytes, dendritic cells (DCs) and activated T cells [53], while immature macrophages, monocytes, DCs or T cells are refractory to releasing sPD-L1 [47]. We speculate that release of sPD-L1 in ovarian cancer patients might be PD-L1^+^M-MDSCs/monocytes/macrophages-dependent. Moreover, sPD-L1 in the blood can be a surrogate marker for expression of blood-circulating PD-L1 on M-MDSCs and monocytes/macrophages. This is clinically valuable because blood tests are relatively simple and non-invasive.

Prognostic relevance of PD-L1 in ovarian cancer is still debatable in the literature and reports are conflicting, whereas some reports highlight no prognostic relevance, other show negative prognostic impact as well as some reports indicate positive prognostic impact of PD-L1 [16–20, 44, 54–58]. In our study cohort we showed that PD-L1 and sPD-L1 were not predictors of OS in patients. Similar, further validation in the independent large cohort of 655 ovarian cancer patients demonstrated no association between PD-L1 mRNA expression and OS. No correlation between PD-L1 protein expression and OS of ovarian cancer patients was also presented in meta-analysis of 1630 ovarian cancers [45]. Due to high heterogeneity of ovarian cancer and different expression profile of PD-L1, these data should be interpreted with caution as PD-L1 expression in some OC subtypes (but not in all OC subtypes) i.e. high grade serous OC [55, 57, 58] and clear cell carcinoma [18] can have prognostic relevance. These discrepancies may also explain disappointing efficacy of checkpoint inhibitors in ovarian cancer (response rate ~ 6–15%) [59]. Unfortunately, due to that our study group did not include OC patients treated with immunotherapy, we are not able to identify whether analyzed PD-L1^+^cells and sPD-L1 can be predictors of immune checkpoint blockade. Indeed, further studies need to be address to stratify OC patients in which PD-L1 on both tumour cells and immune/inflammatory cells may have clinical relevance. Furthermore, to identify of patients in whom immunotherapeutic treatment will be benefit, is crucial.

## Conclusions

To conclude, although data show that PD-L1^+^TT/IC may not be a prognostic marker in ovarian cancer, our study highlight impaired immunity in patients manifested by up-regulation of PD-L1 and sPD-L1. Furthermore, for the first time we revealed positive association between PD-L1^+^myeloid cells and sPD-L1 in the blood, indicate that sPD-L1 may be a noninvasive surrogate marker for surface expression of PD-L1 on myeloid cells in immunomonitoring of ovarian cancer patients. Thus, our findings highlight the relevance of comprehensive analysis of not only TC and IC, but also different population of IC in the different TMEs. Indeed, further validation studies are warranted.

## Supplementary information

**Additional file 1: Fig. S1.** Analysis of monocytic myeloid-derived suppressor cells (M-MDSCs) and monocytes/macrophages (MO/MA) as well as programmed death-ligand 1 (PD-L1)-expressing M-MDSCs and MO/MA in ovarian cancer (OC). Mononuclear cells (MCs) from the blood (n = 43), ascites (n = 26) and tumour tissue (n = 29) of OC patients were analyzed. MCs from the blood of healthy women (n = 15) were also examined. Analysis was performed using flow cytometry. MCs were stained for PD-L1^+^M-MDSCs and MO/MA using fluorochrome-labeled monoclonal antibodies (mAb) against HLA-DR, CD14 and PD-L1(CD274). Representative dot plots from the blood sample of OC patient of HLA-DR^−/low^CD14^+^ M-MDSCs, HLA-DR^+^CD14^+^ MO/MA, PD-L1-expressing M-MDSC and PD-L1-expressing MO/MA are shown.

**Additional file 2: Fig. S2.** Comparative analysis of myeloid cell populations, programmed death-ligand 1 (PD-L1)-expressing myeloid cells and PD-L1 gene expression in the three tumour microenvironments (TMEs) of ovarian cancer (OC) patients. a. Analysis of the percentage of monocytic myeloid-derived suppressor cells (M-MDSCs) and monocytes/macrophages (MO/MA). b. Analysis of the expression profile of PD-L1 on M-MDSCs and MO/MA. c. Expression of PD-L1 in the mononuclear cells (MCs). For all analysis paired samples of blood, ascites and tumour tissue from OC patients were used (n = 10). For PD-L1 gene expression analysis RNA was extracted from the MCs isolated from the blood, ascites and tumour tissue. mRNA expression gene level of PD-L1 was determined using quantitative polymerase chain reaction (qPCR). Data were normalized to the glyceraldehyde 3-phosphate dehydrogenase (GAPDH; fold change). Horizontal lines within the boxes indicate the median and the whiskers indicate the minimum and maximum values.

**Additional file 3: Fig. S3.** Kaplan–Meier graphs with overall survival of ovarian cancer patients a-j. PD-L1 protein expression on immune cells and tumour cells and sPD-L1 concentrations including a. PD-L1^+^M-MDSC in the peripheral blood (n = 43), b. PD-L1^+^MO/MA in the peripheral blood (n = 43), c. PD-L1^+^M-MDSC in the peritoneal fluid (n = 26), d. PD-L1^+^MO/MA in the peritoneal fluid (n = 26), e. PD-L1^+^M-MDSC in the tumour tissue (n = 29), f. PD-L1^+^MO/MA in the tumour tissue (n = 29), g. sPD-L1 in the plasma (n = 39), h. sPD-L1 in the peritoneal fluid (n = 22), i. PD-L1^+^TC (n = 29) and j. PD-L1^+^IC (n = 29); IC-inflammatory/immune cells, M-MDSC - myeloid-derived suppressor cells, MO/MA- monocytes/macrophages, PB-peripheral blood, PD-L1-programmed death-ligand 1, PF-peritoneal fluid, TC-tumour cells, TT-tumour tissue.

**Additional file 4: Fig. S4.** Kaplan–Meier graphs with overall survival of ovarian cancer patients a-h. Microarray datasets (online KM plotter database, JetSet best probe set) were used to validate the results of CD274 (PD-L1) mRNA expression including a. large independent cohort (n = 655) available from all datasets together and from each datasets separately including b. GSE18520 (n = 53), c. GSE19829 (n = 28), d. GSE26193 (n = 107), e. GSE27651 (n = 39), f. GSE30161 (n = 50), g. GSE63885 (n = 25) and h. GSE9891 (n = 285).

## Data Availability

The datasets used during the present study are available from the corresponding author upon reasonable request.

## Abbreviations

ARG1: Arginase 1
DCs: Dendritic cells
ELISA: Enzyme-linked immunosorbent assay
FIGO: The International Federation of Gynecology and Obstetrics
FMO: Fluorescence minus one
GAPDH: Glyceraldehyde 3-phosphate dehydrogenase
HCC: Hepatocellular carcinoma
IHC: Immunohistochemistry
IC: Immune/inflammatory cells
IDO: Indoleamine 2,3-dioxygenase
IL: Interleukin
mAbs: Monoclonal antibodies
MCs: Mononuclear cells
MDSCs: Myeloid-derived suppressor cells
MHC: Major histocompatibility complex
M-MDSCs: Monocytic mdscs
MO/MA: Monocytes/macrophages
NSCLC: Non-small cell lung cancer
OC: Ovarian cancer
OS: Overall survival
PD-L1: Programmed death-ligand 1
qRT-PCR: Quantitative reverse transcription polymerase chain reaction
sPD-L1: Soluble programmed death-ligand 1
TGF-β: Transforming growth factor β
TMEs: Tumour microenvironments
TC: Tumour cells

## References

[1] Lheureux S, Braunstein M, Oza AM (2019). Epithelial ovarian cancer: evolution of management in the era of precision medicine. CA Cancer J Clin.

[2] Okła K, Czerwonka A, Wawruszak A, Bobiński M, Bilska M, Tarkowski R (2019). Clinical relevance and immunosuppressive pattern of circulating and infiltrating subsets of myeloid-derived suppressor cells (MDSCs) in epithelial ovarian cancer. Front Immunol..

[3] Okła K, Wertel I, Polak G, Surówka J, Wawruszak A, Kotarski J (2016). Tumor-associated macrophages and myeloid-derived suppressor cells as immunosuppressive mechanism in ovarian cancer patients: progress and challenges. Int Rev Immunol.

[4] Okla K, Wertel I, Wawruszak A, Bobiński M, Kotarski J (2018). Blood-based analyses of cancer: circulating myeloid-derived suppressor cells—is a new era coming?. Crit Rev Clin Lab Sci..

[5] Drakes M, Stiff P, Drakes ML, Stiff PJ (2018). Regulation of ovarian cancer prognosis by immune cells in the tumor microenvironment. Cancers..

[6] Gabrilovich DI, Ostrand-Rosenberg S, Bronte V (2012). Coordinated regulation of myeloid cells by tumours. Nat Rev Immunol.

[7] Veglia F, Perego M, Gabrilovich D (2018). Myeloid-derived suppressor cells coming of age. Nat Immunol.

[8] Zhu X, Lang J (2017). Soluble PD-1 and PD-L1: predictive and prognostic significance in cancer. Oncotarget..

[9] Kythreotou A, Siddique A, Mauri FA, Bower M, Pinato DJ (2018). PD-L1. J Clin Pathol.

[10] Jiang Y, Zhao X, Fu J, Wang H (2020). Progress and challenges in precise treatment of tumors with PD-1/PD-L1 blockade. Front Immunol..

[11] Stenzel AE, Abrams SI, Moysich KB (2019). Acall for epidemiological research on myeloid-derived suppressor cells in ovarian cancer: a review of the existing immunological evidence and suggestions for moving forward. Front Immunol..

[12] Cui TX, Kryczek I, Zhao L, Zhao E, Kuick R, Roh MH (2013). Myeloid-derived suppressor cells enhance stemness of cancer cells by inducing microRNA101 and suppressing the corepressor CtBP2. Immunity..

[13] Obermajer N, Muthuswamy R, Odunsi K, Edwards RP, Kalinski P (2011). PGE2-induced CXCL12 production and CXCR4 expression controls the accumulation of human MDSCs in ovarian cancer environment. Cancer Res.

[14] Wu L, Deng Z, Peng Y, Han L, Liu J, Wang L (2017). Ascites-derived IL-6 and IL-10 synergistically expand CD14+HLA−DR−/low myeloid-derived suppressor cells in ovarian cancer patients. Oncotarget..

[15] Santegoets SJAM, de Groot AF, Dijkgraaf EM, Simões AMC, van der Noord VE, van Ham JJ (2018). The blood mMDSC to DC ratio is a sensitive and easy to assess independent predictive factor for epithelial ovarian cancer survival. OncoImmunology..

[16] Hamanishi J, Mandai M, Iwasaki M, Okazaki T, Tanaka Y, Yamaguchi K (2007). Programmed cell death 1 ligand 1 and tumor-infiltrating CD8+ T lymphocytes are prognostic factors of human ovarian cancer. Proc Natl Acad Sci USA.

[17] Zhu Y, Zhou S, Liu Y, Zhai L, Sun X (2018). Prognostic value of systemic inflammatory markers in ovarian Cancer: a PRISMA-compliant meta-analysis and systematic review. BMC Cancer..

[18] Zhu J, Wen H, Bi R, Wu Y, Wu X (2017). Prognostic value of programmed death-ligand 1 (PD-L1) expression in ovarian clear cell carcinoma. J Gynecol Oncol..

[19] Mills AM, Peres LC, Meiss A, Ring KL, Modesitt SC, Abbott SE (2019). Targetable immune regulatory molecule expression in high-grade serous ovarian carcinomas in African-American women: a study of PD-L1 and IDO in 112 cases from the African American Cancer Epidemiology Study (AACES). Int J Gynecol Pathol.

[20] Drakes ML, Mehrotra S, Aldulescu M, Potkul RK, Liu Y, Grisoli A (2018). Stratification of ovarian tumor pathology by expression of programmed cell death-1 (PD-1) and PD-ligand- 1 (PD-L1) in ovarian cancer. J Ovarian Res..

[21] Yamauchi Y, Safi S, Blattner C, Rathinasamy A, Umansky L, Juenger S (2018). Circulating and tumor myeloid-derived suppressor cells in resectable non-small cell lung cancer. Am J Respir Crit Care Med.

[22] Kurman RJ, Shih I-M (2016). The dualistic model of ovarian carcinogenesis. Am J Pathol.

[23] Kotsakis A, Harasymczuk M, Schilling B, Georgoulias V, Argiris A, Whiteside TL (2012). Myeloid-derived suppressor cell measurements in fresh and cryopreserved blood samples. J Immunol Methods.

[24] Duffy A, Zhao F, Haile L, Gamrekelashvili J, Fioravanti S, Ma C (2013). Comparative analysis of monocytic and granulocytic myeloid-derived suppressor cell subsets in patients with gastrointestinal malignancies. Cancer Immunol Immunother CII..

[25] Arihara F, Mizukoshi E, Kitahara M, Takata Y, Arai K, Yamashita T (2013). Increase in CD14+HLA-DR−/low myeloid-derived suppressor cells in hepatocellular carcinoma patients and its impact on prognosis. Cancer Immunol Immunother.

[26] Bobiński M, Okła K, Kotarski J, Szumiło J, Polak G, Sobstyl M (2018). Neuropilin 1 in uterine leiomyosarcoma. Clinical and pathological analysis. Ginekol Pol..

[27] Herbst RS, Soria J-C, Kowanetz M, Fine GD, Hamid O, Gordon MS (2014). Predictive correlates of response to the anti-PD-L1 antibody MPDL3280A in cancer patients. Nature.

[28] Gyorffy B, Lánczky A, Szállási Z (2012). Implementing an online tool for genome-wide validation of survival-associated biomarkers in ovarian-cancer using microarray data from 1287 patients. Endocr Relat Cancer.

[29] Buderath P, Schwich E, Jensen C, Horn PA, Kimmig R, Kasimir-Bauer S (2019). Soluble programmed death receptor ligands sPD-L1 and sPD-L2 as liquid biopsy markers for prognosis and platinum response in epithelial ovarian cancer. Front Oncol..

[30] Koukourakis MI, Kontomanolis E, Sotiropoulou M, Mitrakas A, Dafa E, Pouliliou S (2018). Increased soluble PD-L1 levels in the plasma of patients with epithelial ovarian cancer correlate with plasma levels of miR34a and miR200. Anticancer Res.

[31] Pogoda K, Pyszniak M, Rybojad P, Tabarkiewicz J (2016). Monocytic myeloid-derived suppressor cells as a potent suppressor of tumor immunity in non-small cell lung cancer. Oncol Lett..

[32] Rodriguez GM, Galpin KJC, McCloskey CW, Vanderhyden BC. The tumor microenvironment of epithelial ovarian cancer and its influence on response to immunotherapy. Cancers. 2018;10. . 10.3390/cancers10080242 PMC6116043 30042343

[33] Iwata T, Kondo Y, Kimura O, Morosawa T, Fujisaka Y, Umetsu T (2016). PD-L1+ MDSCs are increased in HCC patients and induced by soluble factor in the tumor microenvironment. Sci Rep..

[34] Huang H, Zhang G, Li G, Ma H, Zhang X (2015). Circulating CD14+HLA-DR−/low myeloid-derived suppressor cell is an indicator of poor prognosis in patients with ESCC. Tumor Biol..

[35] Chikamatsu K, Sakakura K, Toyoda M, Takahashi K, Yamamoto T, Masuyama K (2012). Immunosuppressive activity of CD14+HLA-DR− cells in squamous cell carcinoma of the head and neck. Cancer Sci.

[36] Kossaï M, Leary A, Scoazec J-Y, Genestie C (2018). Ovarian cancer: a heterogeneous disease. Pathobiol J Immunopathol Mol Cell Biol..

[37] Wang L, Wang H, Chen H, Wang W, Chen X, Geng Q (2015). Serum levels of soluble programmed death ligand 1 predict treatment response and progression free survival in multiple myeloma. Oncotarget..

[38] Jovanović D, Roksandić-Milenković M, Kotur-Stevuljević J, Ćeriman V, Vukanić I, Samardžić N (2019). Soluble sPD-L1 and serum amyloid A1 as potential biomarkers for lung cancer. J Med Biochem..

[39] Zhang J, Gao J, Li Y, Nie J, Dai L, Hu W (2015). Circulating PD-L1 in NSCLC patients and the correlation between the level of PD-L1 expression and the clinical characteristics. Thorac Cancer..

[40] Chen G, Huang AC, Zhang W, Zhang G, Wu M, Xu W (2018). Exosomal PD-L1 contributes to immunosuppression and is associated with anti-PD-1 response. Nature.

[41] De Nola R, Menga A, Castegna A, Loizzi V, Ranieri G, Cicinelli E, et al. The crowded crosstalk between cancer cells and stromal microenvironment in gynecological malignancies: biological pathways and therapeutic implication. Int J Mol Sci. 2019;20. . 10.3390/ijms20102401 PMC6567055 31096567

[42] Marth C, Wieser V, Tsibulak I, Zeimet AG (2019). Immunotherapy in ovarian cancer: fake news or the real deal?. Int J Gynecol Cancer..

[43] Li C, Li C, Zhi C, Liang W, Wang X, Chen X (2019). Clinical significance of PD-L1 expression in serum-derived exosomes in NSCLC patients. J Transl Med..

[44] Chatterjee J, Dai W, Aziz NHA, Teo PY, Wahba J, Phelps DL (2017). Clinical use of programmed cell death-1 and its ligand expression as discriminatory and predictive markers in ovarian cancer. Clin Cancer Res.

[45] Wang L (2019). Prognostic effect of programmed death-ligand 1 (PD-L1) in ovarian cancer: a systematic review, meta-analysis and bioinformatics study. J Ovarian Res..

[46] Chen Y, Wang Q, Shi B, Xu P, Hu Z, Bai L (2011). Development of a sandwich ELISA for evaluating soluble PD-L1 (CD274) in human sera of different ages as well as supernatants of PD-L1+ cell lines. Cytokine.

[47] Frigola X, Inman BA, Krco CJ, Liu X, Harrington SM, Bulur PA (2012). Soluble B7-H1: differences in production between dendritic cells and T cells. Immunol Lett.

[48] Takahashi N, Iwasa S, Sasaki Y, Shoji H, Honma Y, Takashima A (2016). Serum levels of soluble programmed cell death ligand 1 as a prognostic factor on the first-line treatment of metastatic or recurrent gastric cancer. J Cancer Res Clin Oncol.

[49] Chang B, Huang T, Wei H, Shen L, Zhu D, He W (2019). The correlation and prognostic value of serum levels of soluble programmed death protein 1 (sPD-1) and soluble programmed death-ligand 1 (sPD-L1) in patients with hepatocellular carcinoma. Cancer Immunol Immunother.

[50] Kruger S, Legenstein M-L, Rösgen V, Haas M, Modest DP, Westphalen CB (2017). Serum levels of soluble programmed death protein 1 (sPD-1) and soluble programmed death ligand 1 (sPD-L1) in advanced pancreatic cancer. Oncoimmunology..

[51] Rossille D, Gressier M, Damotte D, Maucort-Boulch D, Pangault C, Semana G (2014). High level of soluble programmed cell death ligand 1 in blood impacts overall survival in aggressive diffuse large B-Cell lymphoma: results from a French multicenter clinical trial. Leukemia.

[52] Ruf M, Moch H, Schraml P (2016). PD-L1 expression is regulated by hypoxia inducible factor in clear cell renal cell carcinoma. Int J Cancer.

[53] Zhang G, Hou J, Shi J, Yu G, Lu B, Zhang X (2008). Soluble CD276 (B7-H3) is released from monocytes, dendritic cells and activated T cells and is detectable in normal human serum. Immunology.

[54] Wang Q, Lou W, Di W, Wu X (2017). Prognostic value of tumor PD-L1 expression combined with CD8+ tumor infiltrating lymphocytes in high grade serous ovarian cancer. Int Immunopharmacol.

[55] Li M, Li H, Liu F, Bi R, Tu X, Chen L (2017). Characterization of ovarian clear cell carcinoma using target drug-based molecular biomarkers: implications for personalized cancer therapy. J Ovarian Res..

[56] Mesnage SJL, Auguste A, Genestie C, Dunant A, Pain E, Drusch F (2017). Neoadjuvant chemotherapy (NACT) increases immune infiltration and programmed death-ligand 1 (PD-L1) expression in epithelial ovarian cancer (EOC). Ann Oncol.

[57] Webb JR, Milne K, Kroeger DR, Nelson BH (2016). PD-L1 expression is associated with tumor-infiltrating T cells and favorable prognosis in high-grade serous ovarian cancer. Gynecol Oncol.

[58] Darb-Esfahani S, Kunze CA, Kulbe H, Sehouli J, Wienert S, Lindner J (2015). Prognostic impact of programmed cell death-1 (PD-1) and PD-ligand 1 (PD-L1) expression in cancer cells and tumor-infiltrating lymphocytes in ovarian high grade serous carcinoma. Oncotarget..

[59] Doo DW, Norian LA, Arend RC (2019). Checkpoint inhibitors in ovarian cancer: a review of preclinical data. Gynecol Oncol Rep..

