# Correction to: Accumulation of blood-circulating PD-L1-expressing M-MDSCs and monocytes/macrophages in pretreatment ovarian cancer patients is associated with soluble PD-L1

**DOI:** 10.1186/s12967-020-02431-8

**Published:** 2020-06-25

**Authors:** Karolina Okła, Alicja Rajtak, Arkadiusz Czerwonka, Marcin Bobiński, Anna Wawruszak, Rafał Tarkowski, Wiesława Bednarek, Justyna Szumiło, Jan Kotarski

**Affiliations:** 1 The First Department of Oncologic Gynecology and Gynecology, Medical University of Lublin, 20-081 Lublin, Poland; 2 Department of Virology and Immunology, Maria Curie-Sklodowska University, 20-031, Lublin, Poland; 3 Department of Biochemistry and Molecular Biology, Medical University of Lublin, 20-081 Lublin, Poland; 4 Department of Clinical Pathomorphology, Medical University of Lublin, 20-090 Lublin, Poland

## Correction to: J Transl Med (2020) 18:220 10.1186/s12967-020-02389-7

Following the publication of the original article [1], it was noted that due to a typesetting error, the Fig. 1 was replaced by a duplicate of Fig. 3. The correct Fig. 1 is given below, and the original article has been corrected.

**Fig. 1:**
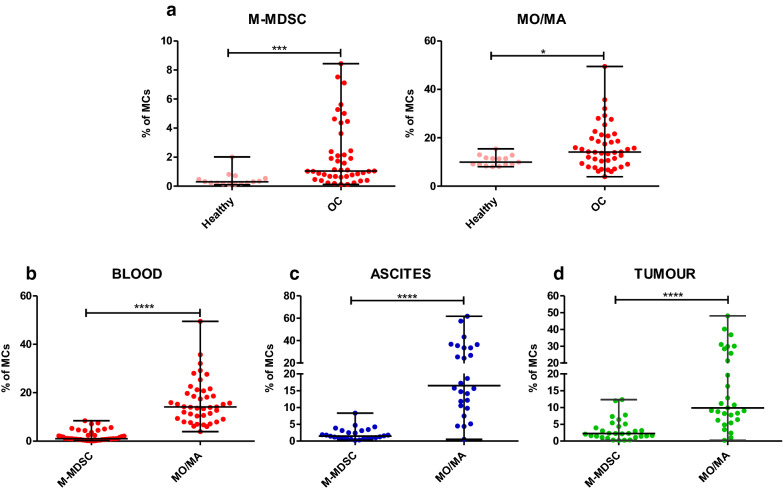
Evaluation of HLA-DR^−/low^CD14^+^ monocytic myeloid-derived suppressor cells (M-MDSCs) and HLA-DR^+^CD14^+^ monocytes/macrophages (MO/MA). Simultaneous analysis of myeloid cell populations in the **a**, **b** blood, **c** ascites and **d** tumour tissue of patients with ovarian cancer. Mononuclear cells (MCs) obtained from the blood (n = 43), ascites (n = 26), and tumour tissue (n = 29) of ovarian cancer patients and from the blood of healthy women (n = 15) were analyzed using flow cytometry. The levels of M-MDSCs and MO/MA are presented as the percentage of MCs. The horizontal lines are the median values and the whiskers indicate the minimum and maximum values. Each point corresponds to an individual patient. *p < 0.05; ***p < 0.001; ****p < 0.0001
