# Supplementary material for: Accumulation of blood-circulating PD-L1-expressing M-MDSCs and monocytes/macrophages in pretreatment ovarian cancer patients is associated with soluble PD-L1
Source: J Transl Med. 2020 Jun 1;18:220. doi: 10.1186/s12967-020-02389-7 (PMC7268341; doi:10.1186/s12967-020-02389-7)
Supplement: Supplementary file 1 — Additional file 1: Fig. S1. Analysis of monocytic myeloid-derived suppressor cells (M-MDSCs) and monocytes/macrophages (MO/MA) as well as programmed death-ligand 1 (PD-L1)-expressing M-MDSCs and MO/MA in ovarian cancer (OC). Mononuclear cells (MCs) from the blood (n = 43), ascites (n = 26) and tumour tissue (n = 29) of OC patients were analyzed. MCs from the blood of healthy women (n = 15) were also examined. Analysis was performed using flow cytometry. MCs were stained for PD-L1+M-MDSCs and MO/MA using fluorochrome-labeled monoclonal antibodies (mAb) against HLA-DR, CD14 and PD-L1(CD274). Representative dot plots from the blood sample of OC patient of HLA-DR−/lowCD14+ M-MDSCs, HLA-DR+CD14+ MO/MA, PD-L1-expressing M-MDSC and PD-L1-expressing MO/MA are shown. [file 12967_2020_2389_MOESM1_ESM.pptx]

## Slide 1
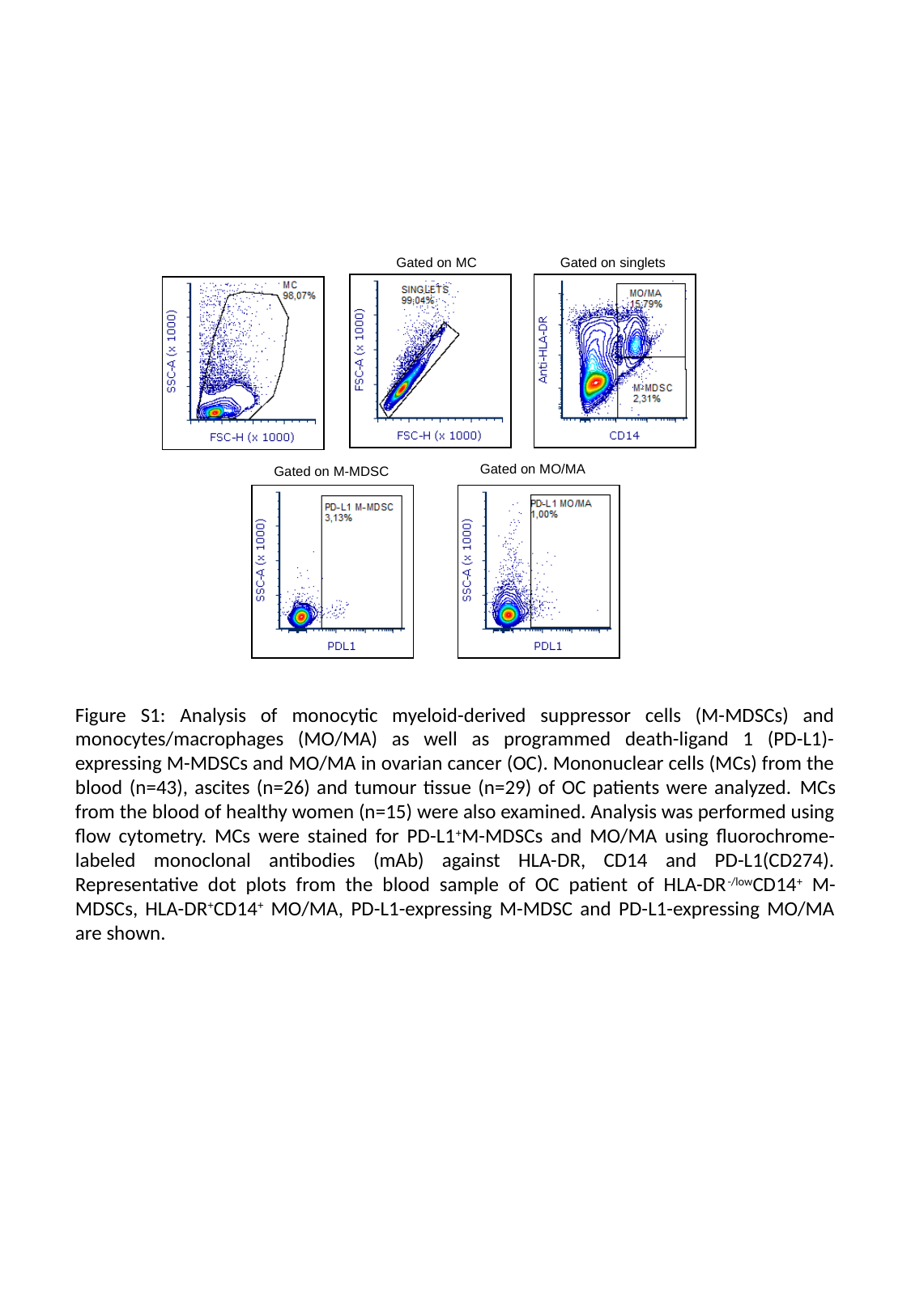

Gated on singlets
Gated on MC
Gated on MO/MA
Gated on M-MDSC
Figure S1: Analysis of monocytic myeloid-derived suppressor cells (M-MDSCs) and monocytes/macrophages (MO/MA) as well as programmed death-ligand 1 (PD-L1)-expressing M-MDSCs and MO/MA in ovarian cancer (OC). Mononuclear cells (MCs) from the blood (n=43), ascites (n=26) and tumour tissue (n=29) of OC patients were analyzed. MCs from the blood of healthy women (n=15) were also examined. Analysis was performed using flow cytometry. MCs were stained for PD-L1+M-MDSCs and MO/MA using fluorochrome-labeled monoclonal antibodies (mAb) against HLA-DR, CD14 and PD-L1(CD274). Representative dot plots from the blood sample of OC patient of HLA-DR-/lowCD14+ M-MDSCs, HLA-DR+CD14+ MO/MA, PD-L1-expressing M-MDSC and PD-L1-expressing MO/MA are shown.
