# Supplementary material for: Accumulation of blood-circulating PD-L1-expressing M-MDSCs and monocytes/macrophages in pretreatment ovarian cancer patients is associated with soluble PD-L1
Source: J Transl Med. 2020 Jun 1;18:220. doi: 10.1186/s12967-020-02389-7 (PMC7268341; doi:10.1186/s12967-020-02389-7)
Supplement: Supplementary file 2 — Additional file 2: Fig. S2. Comparative analysis of myeloid cell populations, programmed death-ligand 1 (PD-L1)-expressing myeloid cells and PD-L1 gene expression in the three tumour microenvironments (TMEs) of ovarian cancer (OC) patients. a. Analysis of the percentage of monocytic myeloid-derived suppressor cells (M-MDSCs) and monocytes/macrophages (MO/MA). b. Analysis of the expression profile of PD-L1 on M-MDSCs and MO/MA. c. Expression of PD-L1 in the mononuclear cells (MCs). For all analysis paired samples of blood, ascites and tumour tissue from OC patients were used (n = 10). For PD-L1 gene expression analysis RNA was extracted from the MCs isolated from the blood, ascites and tumour tissue. mRNA expression gene level of PD-L1 was determined using quantitative polymerase chain reaction (qPCR). Data were normalized to the glyceraldehyde 3-phosphate dehydrogenase (GAPDH; fold change). Horizontal lines within the boxes indicate the median and the whiskers indicate the minimum and maximum values. [file 12967_2020_2389_MOESM2_ESM.pptx]

## Slide 1
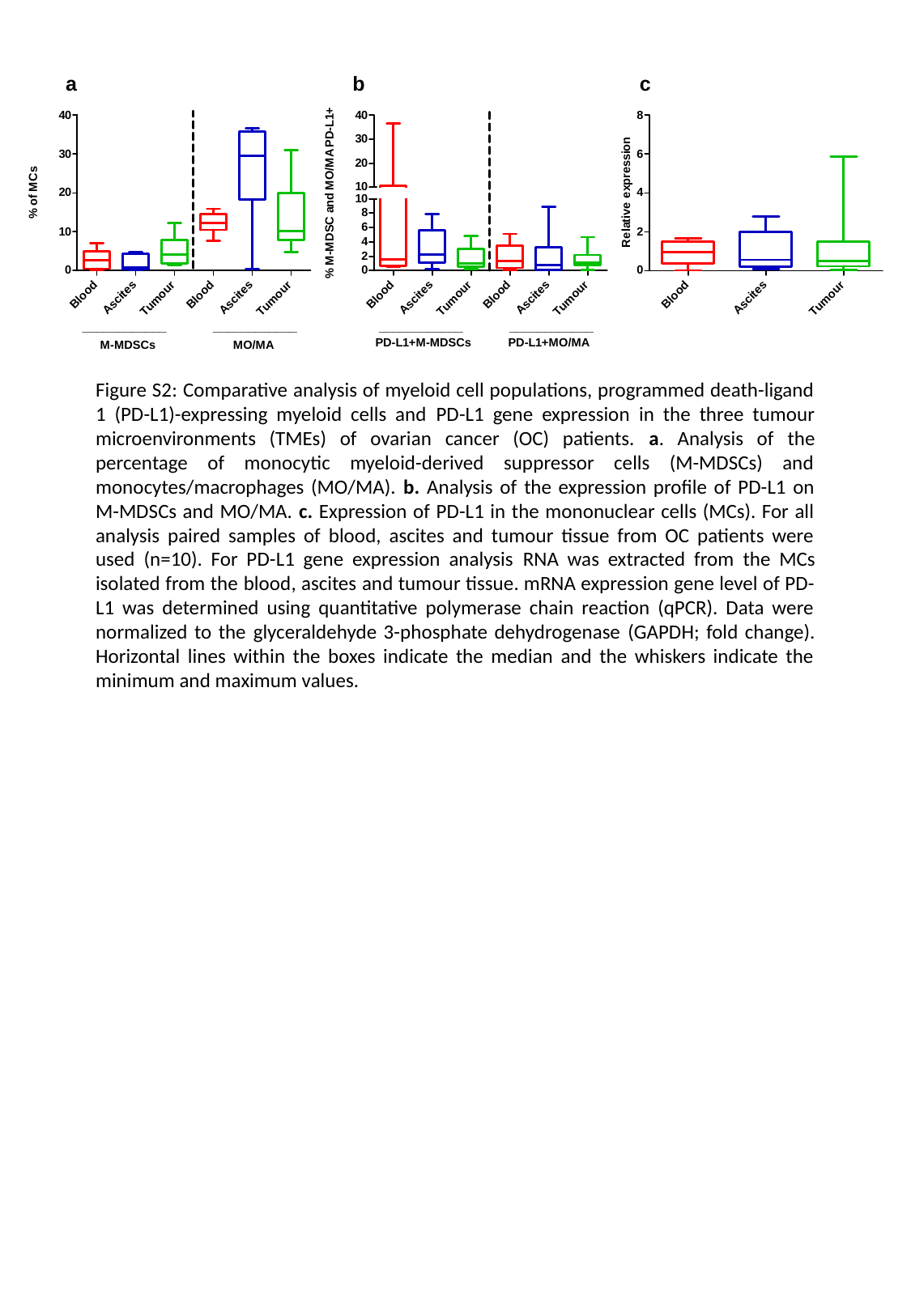

a
b
c
Figure S2: Comparative analysis of myeloid cell populations, programmed death-ligand 1 (PD-L1)-expressing myeloid cells and PD-L1 gene expression in the three tumour microenvironments (TMEs) of ovarian cancer (OC) patients. a. Analysis of the percentage of monocytic myeloid-derived suppressor cells (M-MDSCs) and monocytes/macrophages (MO/MA). b. Analysis of the expression profile of PD-L1 on M-MDSCs and MO/MA. c. Expression of PD-L1 in the mononuclear cells (MCs). For all analysis paired samples of blood, ascites and tumour tissue from OC patients were used (n=10). For PD-L1 gene expression analysis RNA was extracted from the MCs isolated from the blood, ascites and tumour tissue. mRNA expression gene level of PD-L1 was determined using quantitative polymerase chain reaction (qPCR). Data were normalized to the glyceraldehyde 3-phosphate dehydrogenase (GAPDH; fold change). Horizontal lines within the boxes indicate the median and the whiskers indicate the minimum and maximum values.
