# Supplementary material for: Accumulation of blood-circulating PD-L1-expressing M-MDSCs and monocytes/macrophages in pretreatment ovarian cancer patients is associated with soluble PD-L1
Source: J Transl Med. 2020 Jun 1;18:220. doi: 10.1186/s12967-020-02389-7 (PMC7268341; doi:10.1186/s12967-020-02389-7)
Supplement: Supplementary file 3 — Additional file 3: Fig. S3. Kaplan–Meier graphs with overall survival of ovarian cancer patients a-j. PD-L1 protein expression on immune cells and tumour cells and sPD-L1 concentrations including a. PD-L1+M-MDSC in the peripheral blood (n = 43), b. PD-L1+MO/MA in the peripheral blood (n = 43), c. PD-L1+M-MDSC in the peritoneal fluid (n = 26), d. PD-L1+MO/MA in the peritoneal fluid (n = 26), e. PD-L1+M-MDSC in the tumour tissue (n = 29), f. PD-L1+MO/MA in the tumour tissue (n = 29), g. sPD-L1 in the plasma (n = 39), h. sPD-L1 in the peritoneal fluid (n = 22), i. PD-L1+TC (n = 29) and j. PD-L1+IC (n = 29); IC-inflammatory/immune cells, M-MDSC - myeloid-derived suppressor cells, MO/MA- monocytes/macrophages, PB-peripheral blood, PD-L1-programmed death-ligand 1, PF-peritoneal fluid, TC-tumour cells, TT-tumour tissue. [file 12967_2020_2389_MOESM3_ESM.pptx]

## Slide 1
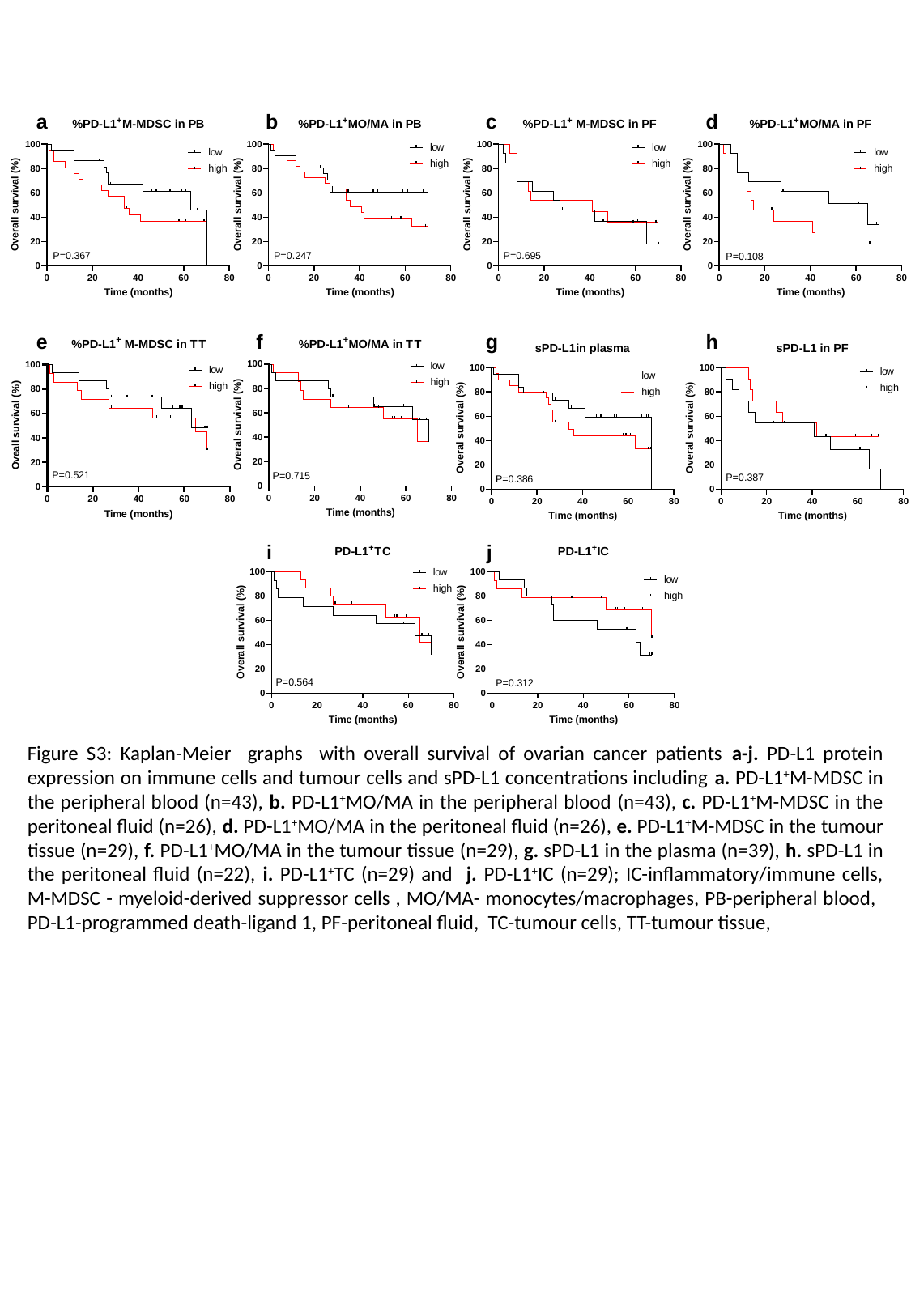

a
b
c
d
e
f
g
h
i
j
Figure S3: Kaplan-Meier graphs with overall survival of ovarian cancer patients a-j. PD-L1 protein expression on immune cells and tumour cells and sPD-L1 concentrations including a. PD-L1+M-MDSC in the peripheral blood (n=43), b. PD-L1+MO/MA in the peripheral blood (n=43), c. PD-L1+M-MDSC in the peritoneal fluid (n=26), d. PD-L1+MO/MA in the peritoneal fluid (n=26), e. PD-L1+M-MDSC in the tumour tissue (n=29), f. PD-L1+MO/MA in the tumour tissue (n=29), g. sPD-L1 in the plasma (n=39), h. sPD-L1 in the peritoneal fluid (n=22), i. PD-L1+TC (n=29) and j. PD-L1+IC (n=29); IC-inflammatory/immune cells, M-MDSC - myeloid-derived suppressor cells , MO/MA- monocytes/macrophages, PB-peripheral blood, PD-L1-programmed death-ligand 1, PF-peritoneal fluid, TC-tumour cells, TT-tumour tissue,
