# Supplementary material for: Accumulation of blood-circulating PD-L1-expressing M-MDSCs and monocytes/macrophages in pretreatment ovarian cancer patients is associated with soluble PD-L1
Source: J Transl Med. 2020 Jun 1;18:220. doi: 10.1186/s12967-020-02389-7 (PMC7268341; doi:10.1186/s12967-020-02389-7)
Supplement: Supplementary file 4 — Additional file 4: Fig. S4. Kaplan–Meier graphs with overall survival of ovarian cancer patients a-h. Microarray datasets (online KM plotter database, JetSet best probe set) were used to validate the results of CD274 (PD-L1) mRNA expression including a. large independent cohort (n = 655) available from all datasets together and from each datasets separately including b. GSE18520 (n = 53), c. GSE19829 (n = 28), d. GSE26193 (n = 107), e. GSE27651 (n = 39), f. GSE30161 (n = 50), g. GSE63885 (n = 25) and h. GSE9891 (n = 285). [file 12967_2020_2389_MOESM4_ESM.pptx]

## Slide 1
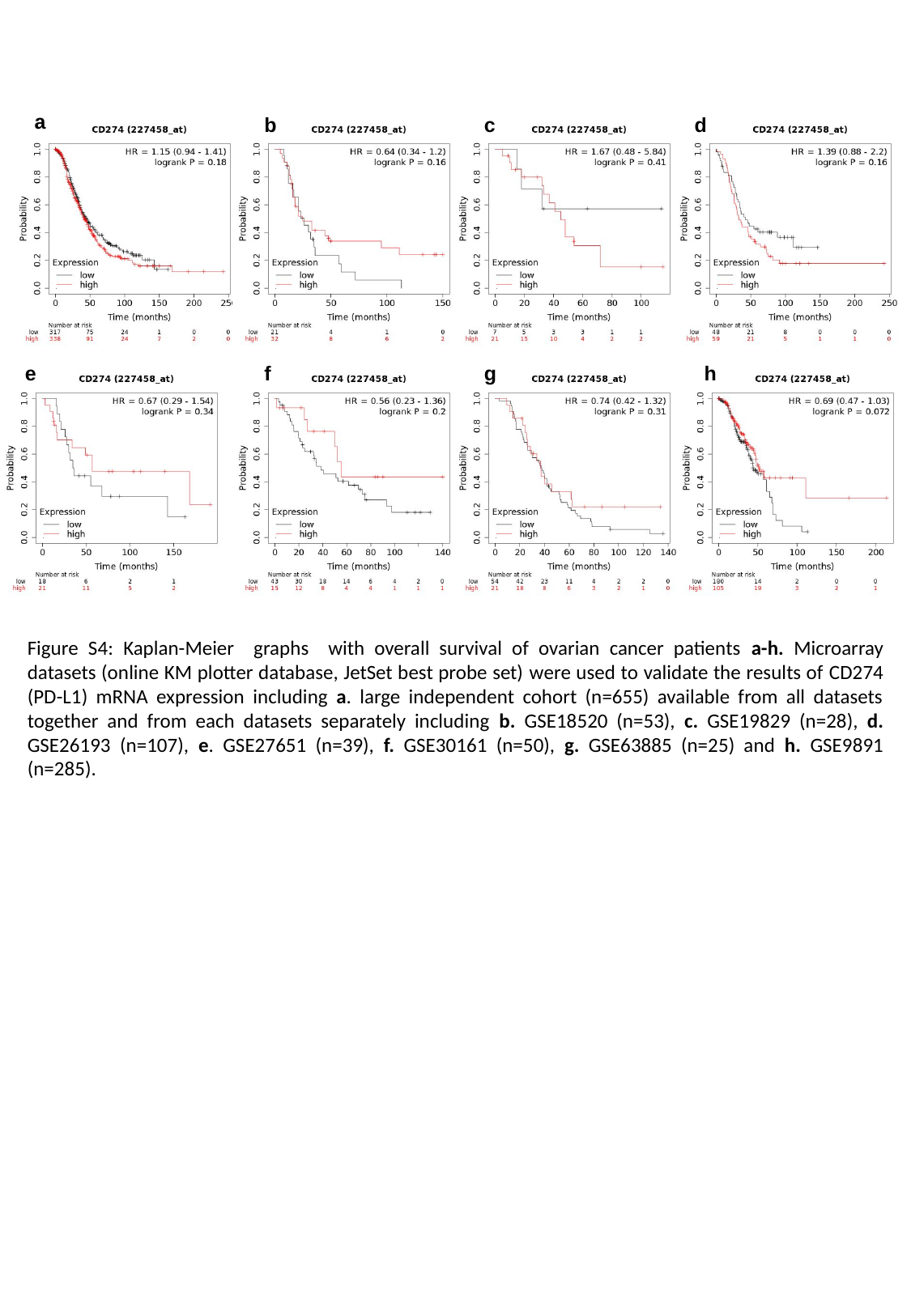

a
b
c
d
e
f
g
h
Figure S4: Kaplan-Meier graphs with overall survival of ovarian cancer patients a-h. Microarray datasets (online KM plotter database, JetSet best probe set) were used to validate the results of CD274 (PD-L1) mRNA expression including a. large independent cohort (n=655) available from all datasets together and from each datasets separately including b. GSE18520 (n=53), c. GSE19829 (n=28), d. GSE26193 (n=107), e. GSE27651 (n=39), f. GSE30161 (n=50), g. GSE63885 (n=25) and h. GSE9891 (n=285).
